# *De novo* PacBio long-read and phased avian genome assemblies correct and add to reference genes generated with intermediate and short reads

**DOI:** 10.1093/gigascience/gix085

**Published:** 2017-08-28

**Authors:** Jonas Korlach, Gregory Gedman, Sarah B. Kingan, Chen-Shan Chin, Jason T. Howard, Jean-Nicolas Audet, Lindsey Cantin, Erich D. Jarvis

**Affiliations:** 1 Pacific Biosciences, Menlo Park, CA 94025, USA; 2 Laboratory of Neurogenetics of Language, Box 54, The Rockefeller University, New York, NY 10065, USA; 3 Department of Biology, McGill University, Montreal, Quebec H3A 1B1, Canada; 4 Howard Hughes Medical Institute, Chevy Chase, MD 20815, USA

**Keywords:** *de novo* genome assembly, long reads, SMRT Sequencing, brain, language

## Abstract

Reference-quality genomes are expected to provide a resource for studying gene structure, function, and evolution. However, often genes of interest are not completely or accurately assembled, leading to unknown errors in analyses or additional cloning efforts for the correct sequences. A promising solution is long-read sequencing. Here we tested PacBio-based long-read sequencing and diploid assembly for potential improvements to the Sanger-based intermediate-read zebra finch reference and Illumina-based short-read Anna's hummingbird reference, 2 vocal learning avian species widely studied in neuroscience and genomics. With DNA of the same individuals used to generate the reference genomes, we generated diploid assemblies with the FALCON-Unzip assembler, resulting in contigs with no gaps in the megabase range, representing 150-fold and 200-fold improvements over the current zebra finch and hummingbird references, respectively. These long-read and phased assemblies corrected and resolved what we discovered to be numerous misassemblies in the references, including missing sequences in gaps, erroneous sequences flanking gaps, base call errors in difficult-to-sequence regions, complex repeat structure errors, and allelic differences between the 2 haplotypes. These improvements were validated by single long-genome and transcriptome reads and resulted for the first time in completely resolved protein-coding genes widely studied in neuroscience and specialized in vocal learning species. These findings demonstrate the impact of long reads, sequencing of previously difficult-to-sequence regions, and phasing of haplotypes on generating the high-quality assemblies necessary for understanding gene structure, function, and evolution.

## Background

Having available genomes of species of interest provides a powerful resource to rapidly conduct investigations on genes of interest. For example, using the original Sanger method to sequence genomes of the 2 most commonly studied bird species, the chicken [1] and zebra finch [2], has impacted many studies. The zebra finch is a vocal learning songbird, with the rare ability to imitate sounds similar to human sounds for speech; comparative analyses of genes in its genome have allowed insights into the mechanisms and evolution of spoken-language in humans [2–4]. With the advent of more cost-effective next-generation sequencing technologies using short reads, 10-fold more vertebrate genomes were sequenced [5], with 1 large successful project being the Avian Phylogenomics Consortium, which generated the genomes of 45 new bird species across the family tree and several reptiles [6, 7]. The consortium was successful in conducting comparative genomics and phylogenomics with populations of genes [8–11]. However, when necessary to dig deeper into individual genes, it was discovered that many were incompletely assembled or contained apparent misassemblies. For example, the *DRD4* dopamine receptor was missing in half of the assemblies, in part due to sequence complexity [12]. The *EGR1* immediate early gene transcription factor, a commonly studied gene in neuroscience and in vocal learning species, was missing the promoter region in a GC-rich region in every bird genome we examined (including the Sanger-based assemblies). Another immediate early gene, *DUSP1*, with specialized vocalizing-driven gene expression in song nuclei of vocal learning species, has microsatellite sequences in the promoters of vocal learning species that are missing or misassembled, requiring single-molecule cloning and sequencing to resolve [13]. Such errors create a great amount of effort to clone, sequence, and correct assemblies of individual genes of interest.

High-throughput, single-molecule, long-read sequencing shows promise to alleviate these problems [14–16]. As part of an effort to evaluate standards for the G10K vertebrate [17] and the B10K bird [18] genome projects, here we applied PacBio single-molecule long-read (1000–60 000 bp) sequencing and diploid assembly on 2 vocal learning species, the zebra finch, previously assembled with Sanger-based intermediate reads (700–1000 bp), and Anna's hummingbird, previously assembled with Illumina-based short reads (100–150 bp). We found that the long-read diploid assemblies resulted in major improvements in genome completeness and contiguity, and completely resolved the problems in all of our genes of interest.

## Results

### The long-read assemblies result in 150-fold to 200-fold increases in contiguity

To generate long-read assemblies, high–molecular weight DNA was isolated from the muscle tissue of the same zebra finch male and Anna's hummingbird female used to create the current reference genomes [2, 8]. The DNA was sheared, 35–40 kb libraries were generated, the DNA was size-selected for inserts >17 kb (Fig. S1), and then SMRT sequencing was performed on the PacBio RS II instrument to obtain ∼×96 coverage for the zebra finch (19-kb N50 read length) and ∼×70 for the hummingbird (22-kb N50 read length) (Fig. S2). The long reads were originally assembled with an early version of the FALCON assembler that only separates very divergent regions between haplotypes and merges the remaining sequence, which we and others found unintentionally introduced indels in the merged regions for some nucleotides that differed between haplotypes (tested on the hummingbird; data not shown) [19]. We then re-assembled using FALCON v. 0.4.0, followed by the FALCON-Unzip module [20] to prevent indel formation and generate longer-range phased haplotypes. Thus, the new assemblies, unlike the current reference assemblies, are phased diploids. This PacBio-based sequencing and assembly approach does not link contigs into gapped scaffolds. Scaffolding requires additional approaches, which we will report on separately in a study comparing scaffolding technologies with these assemblies. The results presented here were found independent of scaffolding.

For the zebra finch, the long-read approach resulted in 1159 primary haplotype contigs with an estimated total genome size of 1.14 Gb (1.2 Gb expected) [21] and contig N50 of 5.81 Mb, representing a 108-fold reduction in the number of contigs and a 150-fold improvement in contiguity compared to the current Sanger-based reference (Table 1A). The diploid assembly process produced 2188 associated, or secondary, haplotype contigs (i.e., haplotigs) with an estimated length of 0.84 Gb and contig N50 of 1.14 Mb (Table 1A), implying that about 75% of the genome contained sufficient heterozygosity to be phased into haplotypes by FALCON-Unzip. Since in FALCON-Unzip, the primary contigs are chosen as the longest path (i.e., longest contig) through the assembly string graph, whether it is from the maternal or paternal chromosome, as the latter information is not known; the secondary haplotigs are thus by definition shorter and greater in number, resulting in lower contiguity for the haplotigs. Regions of the genome with very low heterozygosity remain as collapsed haplotypes in the primary contigs.

**Table 1:** *De novo* genome assembly statistics comparing intermediate-read length and short-read length assemblies with the long-read assemblies

Species	Reference assembly	PacBio-based primary haplotype	Improvement	PacBio-based secondary haplotype
A. Zebra finch	Sanger-based			
Number of contigs	124 806	1159	−108-fold	2188
Contig N50	38 639 bp	5 807 022 bp	+150-fold	2 740 176 bp
Total size	1 232 135 591 bp	1 138 770 338 bp		843 915 757 bp
B. Hummingbird	Illumina-based			
Number of contigs	124 820	1076	−116-fold	4895
Contig N50	26 738 bp	5 366 327 bp	+201-fold	1 073 631 bp
Total size	1 105 676 412 bp	1 007 374 986 bp		1 013 746 550 bp

(A) Zebra finch intermediate-read length (Sanger-based, NCBI accession #GCF_000151805, version 3.2.4) compared to the long-read length PacBio-based assembly. (B) Anna's hummingbird short-read length (Illumina-based, accession #GCF_000699085) compared to the long-read length PacBio-based assembly. Improvement is calculated between the second and third columns for the primary PacBio-based haplotype. The higher number of contigs in the secondary haplotype (fifth column) is a result of the arbitrary assignment of shorter haplotypes to the haplotig category ([18] and main text).

The PacBio long-read assembly for the hummingbird was of similar quality, with 1076 primary contigs generating a primary haploid genome size of 1.01 Gb (1.14 Gb expected) [21], and a contig N50 of 5.36 Mb, representing a 116-fold reduction in the number of contigs and a 201-fold improvement in contiguity over the reference (Table 1B). The length of the assembled secondary haplotigs for the hummingbird was similar to that of the primary contig backbone (1.01 Gb) with a contig N50 of 1.01 Mb (Table 1B) indicating that there was sufficient heterozygosity to phase most of the diploid genome into the 2 haplotypes.

For comparison, using FALCON without the Unzip module [19] resulted in assemblies with high contiguity for the primary contigs (e.g., N50 5.9 Mb for the hummingbird), but much lower for the associated contigs (N50 40 kb). Typical FALCON parameterization allows overlaps between error-corrected reads that differ by ∼5%, and therefore even somewhat divergent haplotypes are collapsed (i.e., merged). Correspondingly, we observed smaller overall associated total assembly sizes (204 Mb for the zebra finch, 187 Mb for the hummingbird, respectively) compared to the more fully phased primary contig assembly sizes (1.11 Gb for the zebra finch, 1.05 Gb for the hummingbird, respectively) (Table 1). The FALCON-Unzip module generates larger haplotigs through phasing of heterozygous single nucleotide polymorphisms (SNPs), and also resolves smaller structural allelic variation. For these reasons, all subsequent analyses were conducted on the more phased FALCON-Unzip assemblies.

### The long-read assemblies have more complete conserved protein coding genes

**Figure 1:**
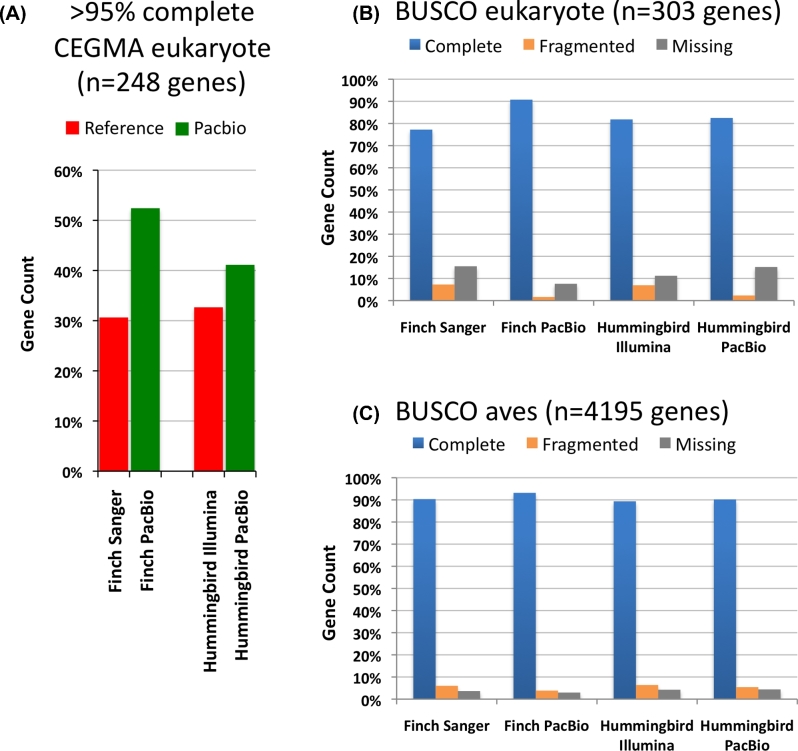
Gene completeness within assemblies. (**A**) Comparison to a 248 highly conserved core CEGMA eukaryote gene set using human genes [23], between the Sanger-based zebra finch and Illumina-based Anna's hummingbird references and their respective PacBio-based assemblies. We used a more stringent cut-off (>95%) for completeness than usually done (>90%) because we felt 90% was too permissive as it could allow entire missing exons and still call a gene as complete. Gene count is the percentage of genes in each of the assemblies that met this criterion. (**B**) Comparison to a 303 single-copy conserved eukaryotic BUSCO gene set [26]. Complete is ≥95% complete; fragmented is <95% complete; missing is not found. (**C**) Comparison to 4915 single-copy conserved genes from the avian BUSCO gene [26].

To assess gene completeness, we analyzed 248 highly conserved eukaryotic genes from the CEGMA human set [22, 23] in each of the assemblies. Both the PacBio-based zebra finch and hummingbird phased assemblies showed improved resolution of these gene sequences, with a close to doubled increase (∼71%) for the zebra finch and a 26% increase for the hummingbird in the number of complete or near-complete (>95%) CEGMA genes assembled, compared to the references (Fig. 1A). Because updating the CEGMA gene sets was recently discontinued due to lack of continued funding and ease of use [24], we also searched for a set of conserved, single-copy genes from the orthoDB9 [25] gene set using the recommended replacement BUSCO pipeline [26]. When assessed using the BUSCO v. 2.0 pipeline on a set of 303 single-copy conserved eukaryotic genes, we observed more modest improvements (∼10%) in the number of complete genes in the zebra finch (and no change with the hummingbird) (Fig. 1B), and barely any change (1–3%) when using a newly generated BUSCO set of 4915 avian genes (Fig. 1C). However, we believe that the moderate increase or no change is due to the fact that much of the BUSCO gene sets were generated from incomplete genome assemblies with short- to intermediate-length reads; for example, the 4915 protein coding avian gene set is generated mostly from the 40+ avian species that the Avian Phylogenomics Project sequenced with short reads [8], including the reference hummingbird [27]. Supporting this view, we extracted the overlapping orthologous genes in the different CEGMA and BUSCO datasets and found that the CEGMA genes are on average significantly longer than their BUSCO counterparts (Fig. S3). When we manually examined randomly chosen genes, many of the BUSCO protein coding sequences were truncated relative to the corresponding CEGMA gene and the PacBio-based assemblies (e.g., the ribosomal protein RLP24 aves BUSCO gene is 117 a.a., whereas the CEGMA and PacBio assembly are 163 a.a.). When compared to the CEGMA 303 eukaryotic set that includes several higher-quality genome assemblies, the PacBio-based assemblies had very few fragmented genes compared to the Sanger-based and Illumina-based assemblies (Fig. 1B). Thus, the new PacBio-based assemblies have the potential to upgrade the BUSCO set with more complete and more accurately assembled genes, a conclusion supported by analyses below.

### The long-read assemblies have greater and more accurate transcriptome and regulome representations

**Figure 2:**
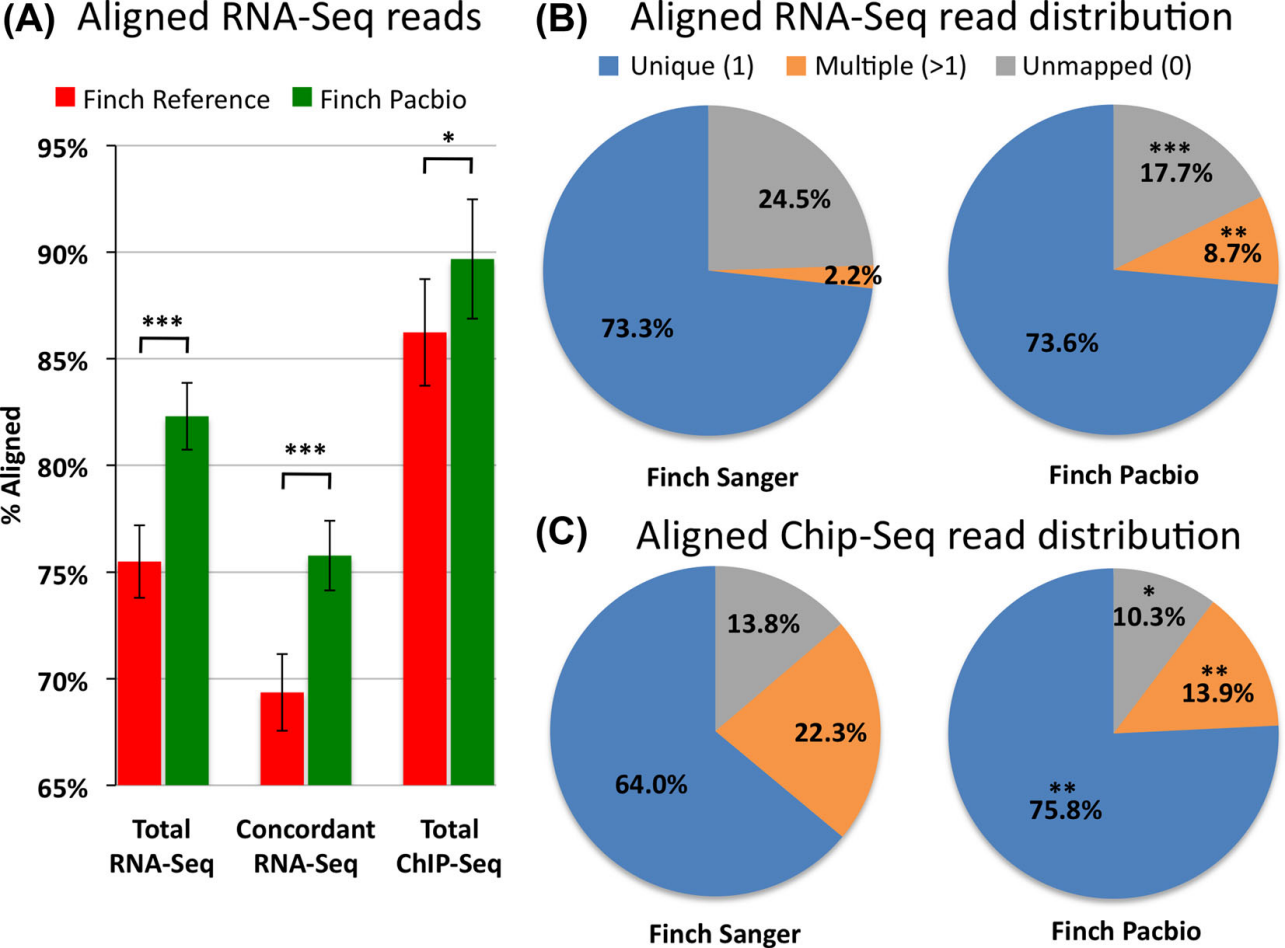
Transcriptome and regulome representation within assemblies. (**A**) Percentage of RNA-Seq and H3K27Ac ChIP-Seq reads from the zebra finch RA song nucleus mapped back to the zebra finch Sanger-based and PacBio-based genome assemblies. (**B**) Pie charts of the distributions of the RNA-Seq reads mapped to the zebra finch genome assemblies. (**C**) Pie charts of the distribution of ChIP-Seq reads mapped to the zebra finch genome assemblies. *P < 0.05; **P < 0.002; ***P < 0.0001; paired *t* test within animals between assemblies; *n* = 5 RNA-Seq and *n* = 3 ChIP-Seq independent replicates from different animals.

To assess transcriptome gene completeness by an approach that does not depend on other species’ genomes, we aligned zebra finch brain paired-end Illumina RNA-Seq reads to the zebra finch genome assemblies using TopHat2 [28]. We generated the RNA-Seq data from microdissected RA song nuclei, a region that has convergent gene expression specialization with the human laryngeal motor cortex (LMC) involved in speech production (Fig. S4) [4]. The PacBio-based assembly (primary haplotype) resulted in a ∼7% increase in total transcript read mappings compared to the Sanger-based reference (Fig. 2A), suggesting more genic regions available for read alignments. This was explained by a decrease in unmapped reads and an increase in reads that mapped to the genome in multiple locations (2 or more) compared to the Sanger-based reference (Fig. 2B), supporting the idea that the long-read assemblies recovered more repetitive or closely related gene orthologs. The PacBio assembly also resulted in ∼6% more concordant aligned paired-end reads (Fig. 2A), indicating a more structurally accurate assembly compared to the Sanger-based reference. RNA-Seq data from the other principle brain song nuclei (HVC, LMAN, and Area X) and adjacent brain regions containing multiple cell types (Fig. S4A) [29] gave very similar results, with 7–11% increased mappings to the PacBio-based assembled genome (not shown).

Regulatory regions have been difficult to identify in the zebra finch genome as they are often GC-rich and hard to sequence and assemble with short-read technologies. To assess the regulome, we aligned HK327ac ChIP-Seq reads generated from the RA song nucleus (see the Methods and [30]) to the zebra finch genome assemblies using Bowtie2 for single-end reads [31]. H3K27ac activity is generally high in active gene regulatory regions, such as promoters and enhancers [32]. Similar to the RNA-Seq transcriptome reads, there was an increase (∼4%) of HK327ac Chip-Seq genomic reads that mapped to the PacBio-based assembly (primary haplotype) compared to the Sanger-based reference (Fig. 2A). However, unlike the RNA-Seq transcript reads, the ChIP-Seq genomic reads showed a significant 10% increase in unique mapped reads with a concomitant decrease in multiple mapped reads (Fig. 2B). We believe this difference is due to technical reasons. The RNA-Seq data were paired-end reads mapped to the genome, whereas the ChIP-Seq data were single-end reads; when just using the single ends of the RNA-Seq data, the multiple-mapped increase to the PacBio-based assembly was not detected (*P* = 0.3, paired *t* test, *n* = 5), indicating that the repetitive sequence in the paired-end data influences read mapping. Overall, these findings are consistent with the PacBio-based assembly having a more complete and structurally accurate assembly for both coding and regulatory non-coding genomic regions.

### Completion and correction of genes important in vocal learning and neuroscience research

The genome-wide analyses above demonstrate improvements to overall genome assembly quality using long reads, but they do not inform about real-life experiences with individual genes. We undertook a detailed analysis of 4 of our favorite genes that have been widely studied in neuroscience and in vocal learning/language research in particular: *EGR1, DUSP1, FOXP2*, and *SLIT1*.

#### EGR1

**Figure 3:**
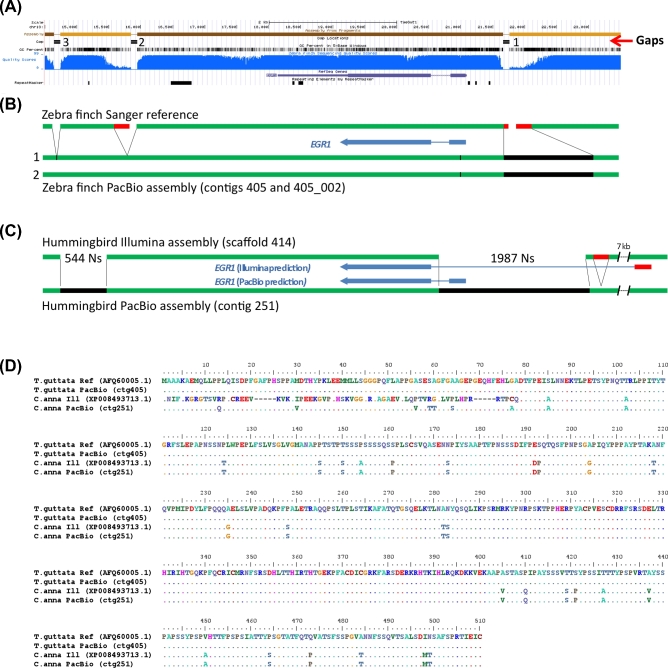
Comparison of *EGR1* assemblies. (**A**) UCSC genome browser view of the Sanger-based zebra finch *EGR1* assembly, highlighting (from top to bottom) 4 contigs (light and dark brown) with 3 gaps, GC percent, nucleotide quality score (blue), RefSeq gene prediction (purple), and areas of repeat sequences. (**B**) Summary comparison of the Sanger-based and PacBio-based zebra finch assemblies, showing in the latter filling the gaps (black) and correcting erroneous reference sequences surrounding the gaps (red). Tick mark is a synonymous heterozygous SNP in the coding region between the primary (1) and secondary (2) haplotypes. Panels *A* and *B* are of the same scale. (**C**) Comparison of the hummingbird Illumina- and PacBio-based assemblies, showing similar corrections that further lead to a correction in the protein coding sequence prediction (blue). (**D**) Multiple sequence alignment of the EGR1 protein for the 4 assemblies (2 zebra finch and 2 hummingbird) in (B) and (C), showing corrections to the Illumina-based hummingbird protein prediction by the PacBio-based assembly.

The early growth response gene 1 (*EGR1*) is an immediate early gene transcription factor whose expression is regulated by activity in neurons and is involved in learning and memory [33]. It is upregulated in song-learning nuclei when vocal learning birds produce song [34]; it belongs to a large set of genes representing 10% of the transcribed genome that are up- or downregulated in response to activity in different cell types of the brain [30]. Studying the mechanisms of regulation of *EGR1* and other immediate early genes has been an intensive area of investigation [35, 36], but in all intermediate- and short-read bird genome assemblies we examined thus far, part of the GC-rich promoter region is missing (Fig. 3A, gap 1).

In the zebra finch Sanger-based reference, *EGR1* is located on a 5.7-kb contig (on chromosome 13), bounded by the gap in the GC-rich promoter region and 2 others downstream of the gene; gaps between contigs in the published reference were given arbitrary 100 Ns [2]. We found that the PacBio long-read assembly resolved all 3 gaps in the *EGR1* locus for both alleles, resulting in complete protein coding and surrounding gene bodies in a 205.5-kb primary contig and a 129.1-kb secondary haplotig (Fig. 3B; Fig. S5A). The promoter region gap was resolved by PacBio-based 804 bp of 70.1% GC-rich sequence (Fig. 3B, black). In addition, to the left and right of this gap, there were 241 bp total of low-quality sequence (<QV40) (Fig 3A, blue, and B, red) that were not supported by the PacBio reads. For the second gap, located ∼2.2 kb downstream of the *EGR1* gene, there was an adjacent 210-bp low-similarity tandem repeat region that also had low-quality scores and was not supported by the PacBio-based reads (Fig 3A and B, gap 2). The third 100 N gap, located ∼3.5 kb downstream of the *EGR1* gene, was resolved by 18 bp of sequence in the PacBio assembly (Fig. 3B, gap 3). The PacBio-based differences in the assembly were supported by numerous long-read (>10 000 bp) molecules that extended through the entire gene, spanning all 3 gaps (Fig. S6A). The 2 haplotypes were >99.8% identical over the region shown (Fig. 3B), with only 1 synonymous heterozygous SNP in the coding sequence (G at position 169 283 in the primary contig 405; T at position 92 478 in secondary haplotig 405_002) (tick mark in Fig. 3B).

In the Illumina-based hummingbird reference, *EGR1* was represented by 3 contigs separated by 2 large gaps of 544 Ns and 1987 Ns, respectively (Fig. 3C), in a large 2.98-Mb scaffold. In contrast, in the PacBio-based hummingbird assembly, *EGR1* was fully resolved in a large 810-kb contig (Fig. 3C). Gene prediction (using Augustus [37]) yielded a protein of the same length as the finch EGR1 protein (510 a.a.), with high (93%) sequence identity (Fig. 3D). The PacBio-based assembly revealed that the larger gap in the Illumina-based assembly harbors the beginning of the *EGR1* gene, including the entire first exon, two-thirds of the first intron, and the GC-rich promoter region (Fig. 3C, black). Due to this gap in the reference, the corresponding NCBI gene prediction (accession XP_008493713.1) instead recruited a stretch of sequence ∼7 kb upstream of the gap, predicting a first exon with no sequence homology to *EGR1* in the PacBio-based assembly or in other species (Fig. 3C and D). Upstream of this gap in the Illumina-based assembly was also a 200-bp tandem repeat that was not supported by the PacBio sequence reads and the assembly (Fig. 3C, red; Fig. S5B). The PacBio-based assembly was further validated by single-molecule Iso-Seq mRNA long-reads of *EGR1* from a closely related species (the ruby-throated hummingbird) [38] that fully contained both predicted exons (Fig. S6B). The PacBio-based assembly did not generate a secondary haplotype for this region, indicating that the 2 alleles are identical or nearly identical for the entire 810-kb contig in the individual sequenced. Upstream and downstream of a high-homology region that includes the *EGR1* gene, there was little sequence homology between the hummingbird and zebra finch assemblies (Fig. S7).

These findings indicate that relative to the intermediate- and short-read assemblies, the PacBio-based long-read assembly can fill in missing gaps in a previously hard-to-sequence GC-rich regulatory region, eliminate low-quality erroneous sequences and base calls at the edges of gaps in the Sanger-based assembly, and eliminate erroneous tandem duplications adjacent to gaps, all preventing inaccurate gene predictions. In addition, using 1 species as a reference to help assemble another may not work for such a gene as the surrounding sequence to the gene body in these 2 Neoaves species is highly divergent.

#### DUSP1

The dual specificity phosphatase 1 (*DUSP1*) is also an immediate early gene, but one that regulates the cellular responses to stress [39]. In all species examined thus far, it is mostly upregulated by activity in the highly active thalamic-recipient primary sensory neurons of the cortex (i.e., mammal cortex layer 4 neurons and the comparable avian intercalated pallial neurons), but within the motor pathways, it is only upregulated to high levels by activity in the vocal learning circuits of vocal learners [13, 40]. This specialized regulation in vocal learning circuits has been proposed to be associated with convergent microsatellite sequences found in the upstream promoter region of the gene mainly in vocal learning species [13]. This was determined by polymerase chain reaction cloning of single genomic molecules from multiple species because the reference assemblies did not have this region properly assembled [13].

**Figure 4:**
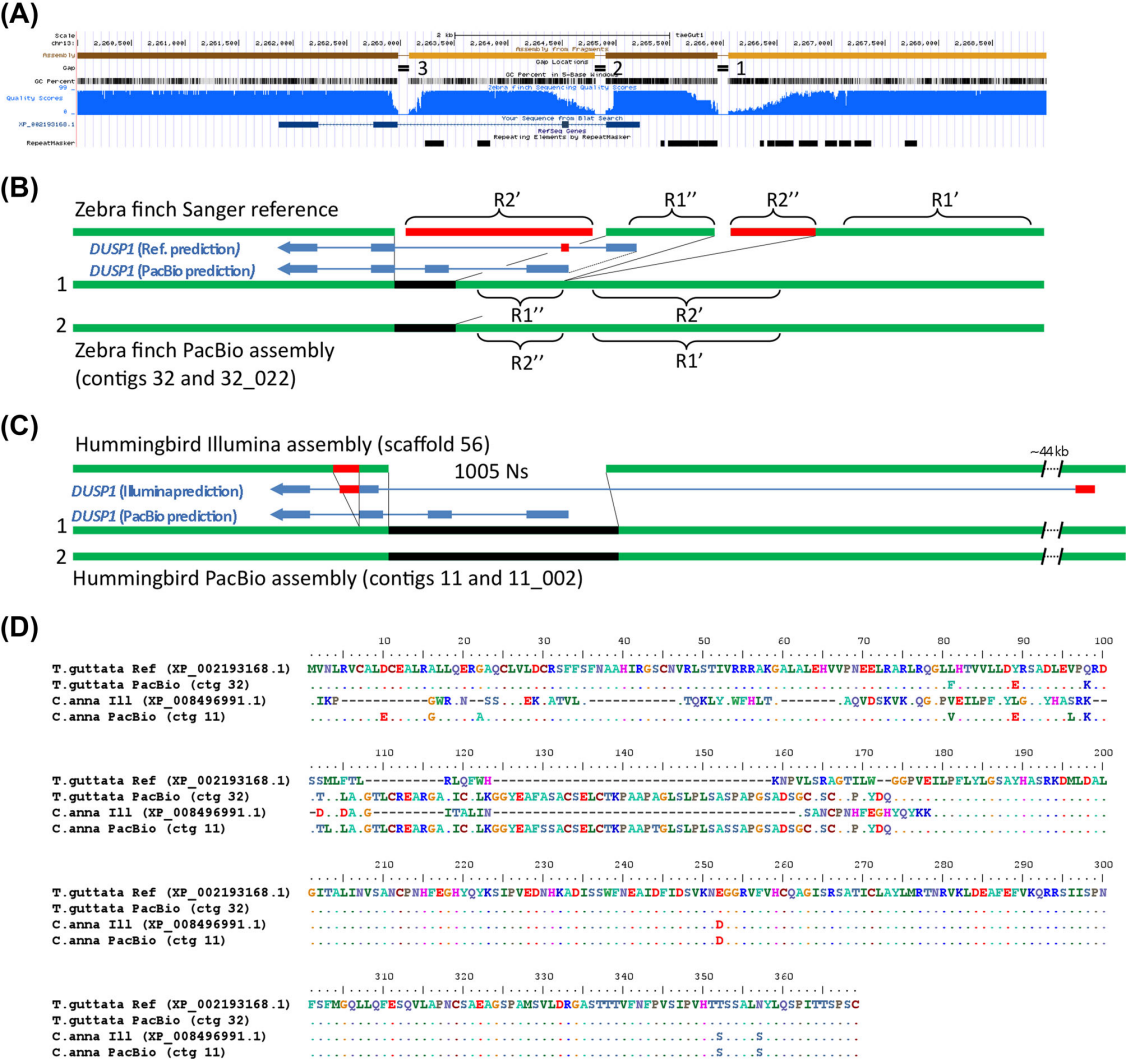
Comparison of *DUSP1* assemblies. (**A**) UCSC genome browser view of the Sanger-based zebra finch *DUSP1* assembly, highlighting 4 contigs with 3 gaps, GC percent, nucleotide quality score, Blat alignment of the NCBI gene prediction (XP_002193168.1, blue), and repeat sequences. (**B**) Resolution of the region by the PacBio-based zebra finch assembly, filling the gaps (black) and correcting erroneous reference sequences in repeat regions (red) and gene predictions (blue). Panels (A) and (B) are of the same scale. (**C**) Resolution and correction to the hummingbird Illumina-based assembly with the PacBio-based assembly (same color scheme as in (B)). (**D**) Multiple sequence alignment of the DUSP1 protein for the 4 assemblies in (B) and (C), showing numerous corrections to the Sanger-based and Illumina-based protein predictions by both PacBio-based assemblies.

In the zebra finch Sanger-based reference, *DUSP1* is located on the chromosome 13 scaffold, separated into 3 contigs, with 2 gaps, all surrounded by low-quality sequences (Fig. 4A). The NCBI gene prediction of this assembly resulted in 4 exons with 322 a.a. (XP_002192168.1), which is ∼13% shorter than *DUSP1* homologs of other species, e.g., chicken (369 a.a., Genbank accession NP_001078828), rat (367 a.a., NP_446221), and human (367 a.a, NP_004408). The 2 gaps coincide with the end of the first predicted exon and the beginning of the third predicted exon (Fig. 4A). An additional gap upstream of the coding sequence falls within the known microsatellite repeat region (Fig. 4A). The PacBio-based assembly produced a completely resolved region for both alleles, in an 8.4-Mb primary contig and an 8.0-Mb secondary haplotig (Fig. 4B, Fig. S8A). The Augustus gene prediction resulted in a protein with 4 exons but now larger, 369 a.a., that was homologous across its length to *DUSP1* of other vertebrate species (e.g., 96% with chicken GGv5 assembly, also recently updated with long reads). Comparing the 2 assemblies revealed that: (i) the first exon in the Sanger-based reference is truncated by 28 a.a. in the gap; (ii) near the edge of that truncation are 3 a.a. that appear to be errors (Fig. 4, residues 81, 89, and 98) as they are different from genomes of other songbird species using high-coverage Illumina reads (Fig. S9A), with strong support in the zebra finch PacBio reads (Fig. S9B); (iii) the second exon and adjacent intron are missing an 80.8% GC-rich 0.46-kb sequence in the reference, which is instead replaced by a 1.7-kb contig of a partially repeated sequence from the microsatellite region upstream of *DUSP1* (R2’ in Fig. 4B), part of which was erroneously recruited in the second exon of the NCBI reference gene prediction (Fig. 4D); and (iv) the microsatellite repeat itself is erroneously partially duplicated in the reference, flanking both sides of gap 1 (R1’’ and R2’’ in Fig. 4B). The PacBio-based phased assembly revealed why both instances of R’ are not identical in the reference: because they in fact belong to the different haplotypes. The 1.7-kb contig corresponds to the upstream region in the primary PacBio haplotype (contig 32) whereas the actual upstream region in the reference corresponds to the upstream region in the secondary PacBio haplotype (contig 32_022) (Fig. 4B). This main microsatellite region is 76 bp longer (796 vs 720 bp) in the primary haplotype, and the neighboring smaller upstream microsatellite contains 3 additional 20–21 bp repeats (11 vs 8) in the primary haplotype (Fig. S10A). Within the protein coding sequence, there were 4 synonymous heterozygous SNPs between haplotypes (not shown). The assembled sequence of the published Sanger-based single clone (AB574425.1) [13] is more consistent with the PacBio-based genome assembly than the Sanger-based reference genome assembly (Fig. S11A) and does not support the erroneous tandem duplications and misplacements of repeat sequences in the latter. Differences in the Sanger-based sequenced clone with the PacBio-based assembly are that the main microsatellite region is smaller (∼320 bp) and the upstream 20–21 bp microsatellite has 10 repeats (instead of 11 or 8), which is consistent with the repeats differing in number between haplotypes (this study) and also individuals [13]. We note that the *DUSP1* haplotypes in the zebra finch PacBio-based FALCON-Unzip assembly are 4.8% divergent, which was below the 5% threshold for allelic segregation in the FALCON assembly without using the Unzip module, but was successfully resolved when using FALCON-Unzip.

In the hummingbird Illumina-based assembly, the *DUSP1* region was represented by 2 contigs separated by a large 1005 N gap (Fig. 4C) on a 7-Mb scaffold. In the PacBio-based assembly, the entire gene was fully resolved (Fig. 4C; Fig. S8B) in a much larger gapless 12.8-Mb contig (the second allele is fully resolved in a 3.8-Mb contig). Comparing the 2 assemblies revealed that the gap of the Illumina-based reference contains about half of the *DUSP1* gene, including the first 2 exons and introns, and ∼380 bp upstream of the start of the gene (Fig. 4C). As a result, the corresponding NCBI gene prediction (XP_008496991.1) recruited a sequence ∼44 kb upstream predicting 46 a.a. with no sequence homology to *DUSP1* of other species, whereas the PacBio-based assembly yielded a 369 a.a. protein with 99% sequence identity to the PacBio-based zebra finch and chicken *DUSP1* (Fig. 4D). A 200-bp tandem repeat in the Illumina-based assembly downstream of the gap, erroneously in exon 3, is a misplaced copy of the microsatellite region (Fig. 4C; Fig. S8B). This is the reason why two-thirds of exon 3 is erroneously duplicated in the NCBI protein prediction (Fig. 4D). These differences in the PacBio-based assembly were validated by single-molecule Iso-Seq mRNA long reads (Fig. S12A) and a Sanger-based assembly of a single clone (AB574427.1; Fig. S11B) of *DUSP1*. The PacBio assemblies also revealed that the microsatellite region was significantly shorter in the hummingbird (∼270 bp) than in the zebra finch genome (∼1100 bp; Fig. S10B).

These findings in both species demonstrate that intermediate- and short-read assemblies not only have gaps with missing relevant repetitive microsatellite sequence, but that short-read misassemblies of these repetitive sequences lead to erroneous protein coding sequence predictions. Further, not only does the long-read assembly resolve them, but it helps generate a diploid assembly that resolves allelic differences and prevents erroneous assembly duplications and misplacement errors between haplotypes.

#### FOXP2

**Figure 5:**
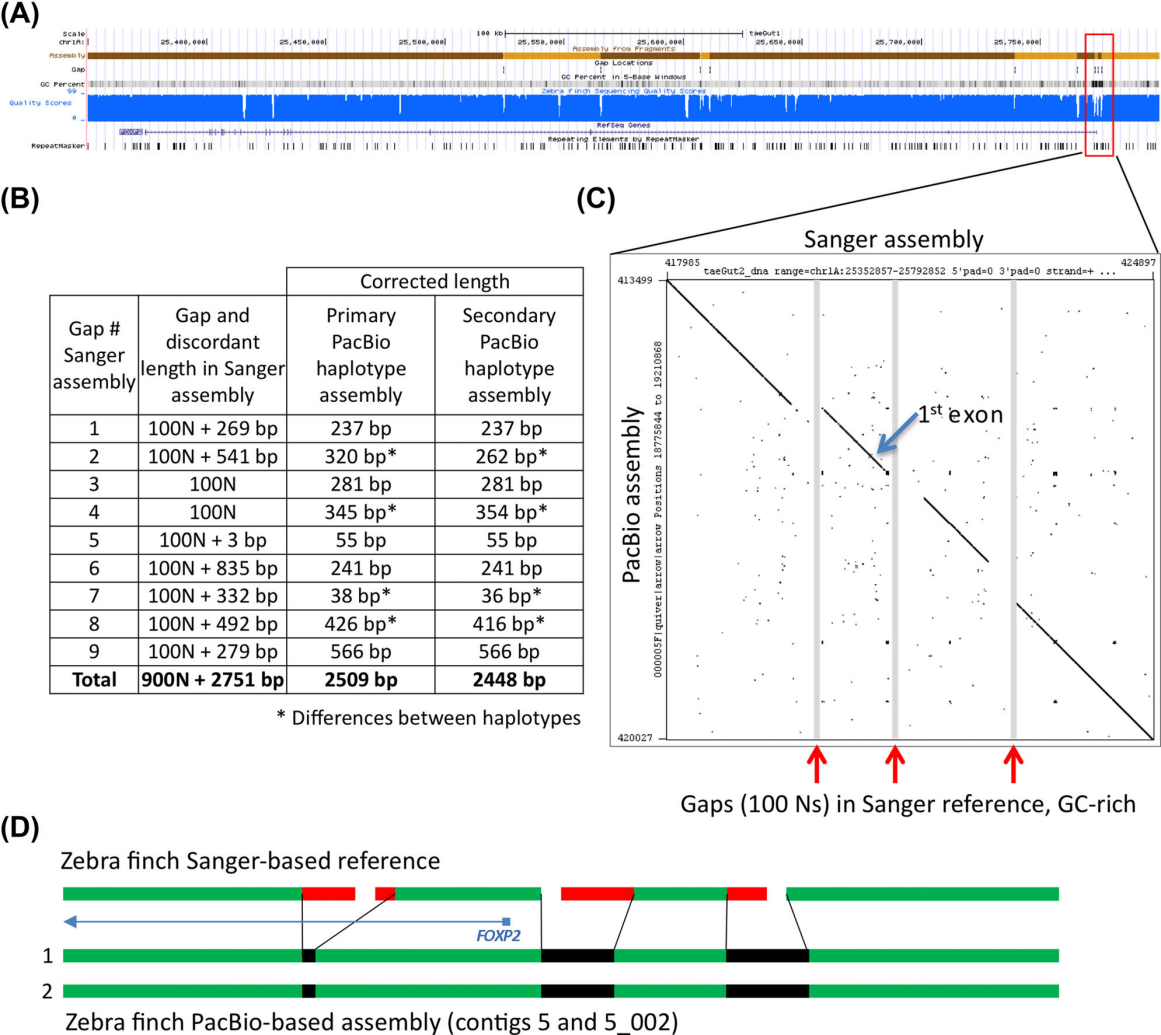
Comparison of *FOXP2* assemblies. (**A**) UCSC genome browser view of the Sanger-based zebra finch *FOXP2* assembly, highlighting 10 contigs with nine gaps, GC percent, nucleotide quality score, RefSeq gene prediction, and repeat sequences. (**B**) Table showing the number of resolved and corrected erroneous base pairs in the gaps by the PacBio-based primary and secondary haplotype assemblies; the asterisk indicates differences between haplotypes. (**C**) Dot plot of the Sanger-based reference (x-axis) and the PacBio-based primary assembly (y-axis) corresponding to the 3 GC-rich region gaps immediately upstream and surrounding the first exon of the *FOXP2* gene. (**D**) Schematic summary of corrections to the 3 gaps shown in (C) in the 2 haplotypes of the PacBio-based assembly. The protein coding sequence alignments are in Figure S13A.

The forkhead box P2 (*FOXP2*) gene plays an important role in spoken language acquisition [41]. In humans, a point mutation in the protein coding binding domain in the KE family [42], as well as deletions in the non-coding region of *FOXP2* [43], results in severe spoken language impairments in heterozygous individuals (homozygous is lethal). In songbirds, FOXP2 expression in the Area × song nucleus is differentially regulated by singing activity and during the song learning critical period, and is necessary to properly imitate song [44–46]. In mice, although vocalizations are mainly innate, animals with the KE mutation demonstrate a syntax apraxia-like deficit in syllable sequencing similar to that of humans [47, 48]. Thus, *FOXP2* has become the most studied gene for understanding the genetic mechanisms and evolution of spoken language [49], yet we find that the very large gene body of ∼400 kb is incompletely assembled (Fig. 5A).

In the zebra finch Sanger-based reference, *FOXP2* is located on the chromosome 1A scaffold and separated into 10 contigs (1 to 231 kb in length) with nine 100-N gaps (Fig. 5A). These include 2 gaps immediately upstream of the first exon, making the beginning of the gene poorly resolved. The provisional RefSeq mRNA for *FOXP2* (NM_001048263.1) contains 19 exons and encodes a 711-a.a. protein (NP_001041728.1). In the PacBio-based assembly, the entire 400-kb gene is fully resolved for both haplotypes in 21.5 Mb and 7.6 Mb contigs, respectively (Fig. S13A). As observed in the previous examples, low-quality sequences of various sizes surrounding all 9 gaps in the Sanger-based reference were unsupported by the PacBio higher-quality data, resulting in a total of 2509 bp of corrected sequence in the PacBio-based primary haplotype (Fig. 5B). The 2 filled gaps in the upstream region and the next gap in the first intron were GC-rich (77.6%, 66.5%, and 67.8%, respectively) (Fig. 5A and C), indicative of the likely cause of the poor-quality Sanger-based read coverage (Fig. 5D). The DNA sequence between the 2 assembled PacBio haplotypes was >99% similar across the entire 400-kb *FOXP2* gene and identical over the coding sequence, with differences occurring in the more complex non-coding gaps that were difficult to sequence and assemble by the Sanger method (Fig. 5B *61 nucleotide differences total). The predicted protein sequence from the PacBio-based assembly is identical to the predicted Sanger-based reference (NP_001041728.1), with the exception of a.a. residue 42 (threonine vs serine) (Fig. S14A). The PacBio nucleotide call also exists in the mRNA sequence of another zebra finch animal in NCBI (NM_001048263.2) and in other avian species we examined, and is thus likely a base call error in the Sanger-based zebra finch reference.

In the hummingbird Illumina-based assembly, as expected with short-read assemblies relative to the Sanger-based zebra finch reference, the *FOXP2* gene was even more fragmented, in 23 contigs (ranging from 0.025 to 2.28 kb in length) with 22 gaps (Fig. S13B). The 2 largest gaps encompass the beginning of the gene and first (non-coding) exon, resulting in corresponding low-quality predicted mRNA (XM_008496149.1). The predicted protein (XP_008494371.1) includes an introduced correction (a.a. 402) (Fig. S14A, X nucleotide) to account for a genomic stop codon, and an 88-N gap within exon 6 that artificially splits the exon into 2 pieces (Fig. S14B). In the hummingbird PacBio-based assembly, the *FOXP2* gene is fully resolved and phased into 2 haplotype contigs of 3.2 Mb each (Fig. S13B). The erroneous stop codon is corrected (2170128C [ctg 110] and 2183088C [ctg 110_009], instead of 841788T [Illumina assembly scaffold 125]), and exon 6 is accurately contiguous, removing the gap and an additional 22 bp of erroneous tandem repeat sequence adjacent to the gap (Fig. S14B and C). The PacBio-based assembly also corrects 3 other instances of erroneous tandem duplications over the gene region in the Illumina-based assembly, as well as removes a 462-bp stretch of sequence adjacent to a long homonucleotide A stretch in intron 1 of the Illumina-based assembly (position 972 040) (Fig. S15A). These PacBio-based differences in the assembly were validated by single-molecule Iso-Seq mRNA long reads of *FOXP2* (Fig. S12B). The 2 PacBio assembled haplotypes are >99% similar, with 1 heterozygous SNP (2172601T (contig 110) vs 2185560A (contig 110_009)) in exon 6 that is silent and a 708-bp deletion in the secondary haplotype (contig 110_009 [at position 2 128 952] relative to contig 110) (Fig. S15B). The Illumina-based assembly has the deleted allele.

These findings replicate those of the previously discussed genes, and in addition show that the PacBio-based assembly can fully resolve very large genes, resolve erroneous assembled sequences in gaps due to repeats or homonucleotide stretches, and reveal large haplotype differences. The phased diploid assembly also avoids the possibility of large missed sequences in a haploid-only assembly due to deletions in one allele.

#### SLIT1

Slit homolog 1 (*SLIT1*) is a repulsive axon guidance ligand for the *ROBO1* receptor and is involved in circuit formation in the developing brain [50]. Recently, *SLIT1* was shown to have convergent specialized downregulated expression compared to the surrounding brain region in the RA song nucleus of all independently evolved vocal learning bird lineages and in the analogous human LMC (Fig. S4) [4, 51], indicating a potential role of *SLIT1* in the evolution and formation of vocal learning brain circuits. A fully resolved *SLIT1*, including regulatory regions, is necessary to assess the mechanisms of its specialized regulation in vocal learning brain regions.

**Figure 6:**
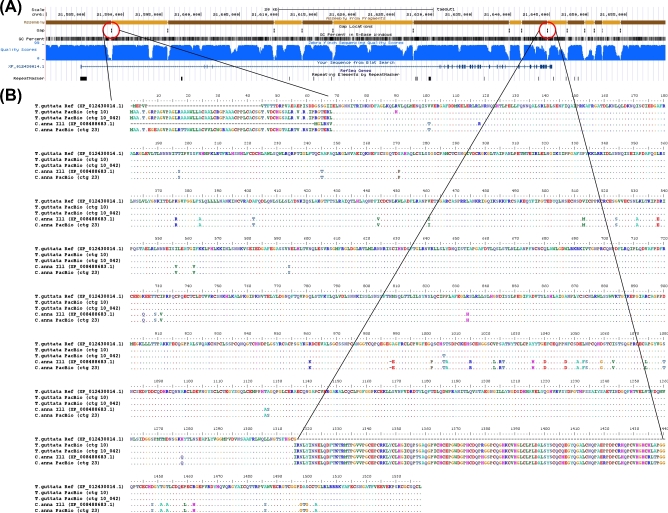
Comparison of *SLIT1* assemblies. (**A**) UCSC genome browser view of the Sanger-based zebra finch *SLIT1* assembly, highlighting 15 contigs with 14 gaps, GC percent, nucleotide quality score, NCBI *SLIT1* gene prediction (XP_012430014.1, blue), and repeat sequences. Red circles are gaps that correspond to the missing exon 1 and part of the missing exon 35, respectively. (**B**) Multiple sequence alignment comparison of the SLIT1 protein for the 4 assemblies compared, including the 2 different haplotypes from the PacBio-based zebra finch assembly (rows 2 and 3).

In the zebra finch Sanger-based reference, *SLIT1* is located on chromosome 6, split among 8 contigs with 7 gaps, and 7 additional contigs and gaps surrounding the ∼40-kb gene (Fig. 6A). The *SLIT1* gene is complex, with over 35 exons. We noted an incomplete predicted protein of the reference (XP_012430014.1) relative to some other species (chicken [NM_001277336.1], human [NM_003061.2], and mouse [NM_015748.3]); our *de novo* gene predictions of the reference also resulted in a truncated protein with 2 missing exons (Fig. 6B). The PacBio-based assembly fully resolved and phased the gene region, in 2 alleles on 15.7-Mb and 5.6-Mb contigs, respectively, and completely recovered all 35+ exons (Fig. S16A). Similar to above, reference sequences flanking the gaps were found to be erroneous and corrected, and an erroneous tandem duplication was also corrected (not shown). Filling in these gaps recovered the 2 missing exons: exon 1 within a 1-kb region of sequence in the PacBio-based assembly that is 75% GC-rich, replacing 390 bp of erroneous gap-flanking sequence, and exon 35 adjacent to a gap (Fig. 6A, B). A predicted exon upstream of exon 1 in a repeat region was not supported (Fig. 6A, B). The gene is heterozygous in the individual, with 3 codon differences between the 2 alleles (Fig. 6B, positions 90, 1006, and 1363, respectively), and an additional 24 silent heterozygous SNPs across the coding region.

In the hummingbird Illumina-based assembly, the *SLIT1* gene is separated on 9 contigs with 8 gaps ranging in length from 91 to 1018 bp, comprising 3320 bp of missing sequence, or 5.3% of the gene region (Fig. S16B). The PacBio-based assembly fully resolved and phased *SLIT1* into haplotypes on 9.9-Mb contigs (Fig. S16B). The resulting protein of 1538 a.a. has high sequence identity to the zebra finch PacBio-based *SLIT1* (95% a.a. identity) (Fig. 6B), and the individual is homozygous for the SLIT1 protein. Comparisons revealed that, as with the Sanger-based reference, the first exon (68 a.a.) is missing completely in the Illumina-based assembly (Fig. 6B), corresponding to a gap of 495 Ns, which the PacBio-based assembly replaced by a 567-bp 76% GC-rich sequence (Fig. S16B). In addition, there were 2 sequence errors in the Illumina-based assembly that were not found in the PacBio-based assembly or Sanger-based assemblies of other species, which resulted in erroneous amino acid predictions in the SLIT1 protein (Fig. 6B, positions 118 and 1381, respectively).

These findings demonstrate that long-read assemblies can fully resolve a complex multi-exon gene, as well as have a higher base call accuracy than Sanger- or Illumina-based reads in difficult-to-sequence regions, including exons, leading to higher protein-coding sequence accuracy.

#### Other genes

We have manually compared several dozen other genes between the different assemblies and found, in all cases investigated, errors in the Sanger-based and Illumina-based assemblies that were prevented in the PacBio-based long-read assemblies. These genes included other immediate early gene transcription factors, other genes in the *SLIT* and *ROBO* gene families, and the *SAP30* gene family. All had the same types of errors in the genes discussed above. In addition, we also found cases where genes were missing from the Sanger-based zebra finch or Illumina-based hummingbird assemblies entirely, and could have been interpreted as lost in these species. These included the DNA methyltransferase enzyme *DNMT3A* missing in the Sanger-based finch assembly and *DRD4* missing in the hummingbird assembly [12], with both fully represented in the PacBio-based assemblies. We also noted cases where an assembled gene was incorrectly localized on a scaffold in the Sanger-based assembly whose synteny was corrected with the PacBio-based assembly, such as the vasopressin receptor AVPR1B, which will be reported on in more detail separately. Data for these types of errors were not shown due to space limitations, but they offer further examples of the important improvements of PacBio long-read technology for generating more accurate genome assemblies.

## Discussion and Conclusions

Although the intermediate-read and short-read assemblies had a high proportion of correct sequences and assembled regions in terms of total base pairs covered, the long-read assemblies revealed numerous errors within and surrounding many genes. These errors are not simply in so-called “junk” intergenic repetitive DNA known to be hard to assemble with short reads [52, 53], but within functional regions of genes. Table 2 summarizes 10 broad categories of errors we found, including gaps, base call errors, gene prediction errors, and missing genes, and assigns which of the 3 main improvements prevented them in our *de novo* assemblies: long reads, SMRT sequencing reading through normally difficult-to-sequence regions, and phasing of haplotypes. Some compounded errors include the assemblers for the short reads sometimes erroneously inserting a repetitive sequence in a non-repetitive region of a gene. These and other assembly and sequence errors and gaps in the sequences can all lead to gene and protein coding sequence prediction errors.

**Table 2:** Summary of error types found in the different sequencing/assembly approaches and the 3 main factors that improved them in the *de novo* assemblies and gene predictions presented in this study

Error type	Caused by	Sequence and/or assembly approach	Improved by
Gaps	Difficult to sequence, assembly algorithm errors with short reads	Sanger- & Illumina-based	Long reads
Low-quality sequences surrounding gaps	Low coverage of difficult to sequence GC-rich & other seq	Sanger- & Illumina-based	SMRT sequencing read through
Base call errors	Difficult to sequence GC-rich & other seq	Sanger- & Illumina-based	SMRT sequencing read through
Tandem, microsatellite, and other repeat errors	Difficult to assemble with short reads	Sanger- & Illumina-based	Long reads
Homonucleotide stretch assembly errors	Misassembly with short reads	Illumina-based	Long reads
InDel errors	Assembly algorithm	PacBio-based	Phasing
Misplaced/merged haplotype errors	Assembly algorithm	Sanger-, Illumina-, & PacBio-based unphased	Long reads and phasing
Gene prediction errors	All errors above and haplotype merging errors	Sanger-, Illumina-, and PacBio-based unphased	Long reads, phasing, and coverage
Missing gene errors	Short & intermediate raw reads not able to be assembled	Sanger- & Illumina-based	Long reads
Misplaced gene synteny	Insufficient sequence data around paralogous genes	Sanger- & Illumina-based approach	Long reads

The long-read, phased assemblies prevented these problems and for the first time resolved gene bodies of all the genes we examined into single, contiguous, gap-less sequences. The phasing of haplotypes, although initially done to prevent a computationally introduced indel error, reveal how important phasing is to prevent assembly and gene prediction errors. Thus far, we have not seen an error (i.e., difference) in the genes we examined in the PacBio-based long-read, phased assemblies relative to the other assemblies, with orthogonal support from both PacBio-based datasets (single sequenced genomic DNA molecules, Iso-Seq mRNA molecules) and other independent evidence (Illumina RNA-Seq and Sanger single clone data). With these improvements, we now, for the first time, have complete and accurate assembled genes of interest that can be pursued further without the need to individually and arduously clone, sequence, and correct the assemblies one gene at a time.

Our study also highlights the value of maintaining frozen tissue or cells of the individuals used to create previous reference genomes as we could only discover some of the errors (e.g., caused by haplotype differences) by long-read *de novo* genome assemblies of the same individual used to create the reference. We are now using these PacBio-based assemblies with several groups and companies as starting assemblies for scaffolding into phased, diploid, chromosome-level zebra finch and hummingbird assemblies to upgrade the references, which will be reported on separately. However, even without scaffolding, these more highly contiguous assemblies will be helpful to researchers to extract more accurate assemblies of their genes of interest, saving a great amount of time and energy, while adding new knowledge and biological insights necessary for understanding gene structure, function, and evolution.

## Materials and Methods

### DNA isolation

For both the zebra finch and hummingbird, frozen muscle tissue from the same animals used to create the Sanger-based [2] and Illumina-based [8] references, respectively, was processed for DNA isolation using the KingFisher Cell and Tissue DNA Kit (97 030 196). Tissue was homogenized in 1 ml of lysis buffer in M tubes (Miltenyi Biotec) using the gentleMACS™ Dissociator at the Brain 2.01 setting for 1 minute. The cell lysate was treated with 40 ul of protease K (20 mg/ml) and incubated overnight. DNA was purified using the KingFisher Duo system (5 400 100) using the built-in KFDuoC_T24 DW program.

### Library preparation and sequencing

For the zebra finch, 2 samples were used for library construction. Each DNA sample was mechanically sheared to 60 kb using the Megaruptor system (Diagenode). Then >30-kb libraries were created using the SMRTbell Template Prep Kit 1.0 (Pacific Biosciences), which includes a DNA Damage Repair step after size selection. Size selection was made for 15 kb for the first sample and 20 kb for the second sample, using a Blue Pippin instrument (Sage Science) according to the protocol “Procedure & Checklist – 20 kb Template Preparation Using BluePippin Size-Selection System.” For the hummingbird, 70 ug of input DNA was mechanically sheared to 35 and 40 kb using the Megaruptor system, a SMRTbell library was constructed, and DNA was size-selected to >17 kb with the BluePippin. Library quality and quantity were assessed using the Pippin Pulse field inversion gel electrophoresis system (Sage Science), as well as with the dsDNA Broad Range Assay kit and Qubit Fluorometer (Thermo Fisher).

SMRT sequencing was performed on the Pacific Biosciences RS II instrument at Pacific Biosciences using an on-plate concentration of 125 pM, P6-C4 sequencing chemistry, with magnetic bead loading, and 360-minute movies. A total of 124 SMRT Cells were run for the zebra finch, and 63 SMRT Cells for the hummingbird. Sequence coverage for the zebra finch was ∼96-fold, with half of the 114 Gb of data contained in reads longer than 19 kb. For the hummingbird, coverage was ∼70-fold, with half of the 40.4 Gb of data contained in reads longer than 22 kb (Fig. S2).

### Assembly

Assemblies were carried out using FALCON v. 0.4.0 followed by the FALCON-Unzip module [20]. FALCON is based on a hierarchical genome assembly process [54]. It constructs a string graph from error-corrected PacBio reads that contain “haplotype-fused” genomic regions as well as “bubbles” that capture divergent haplotypes from homologous genomic regions. The FALCON-Unzip module then assigns reads to haplotypes using heterozygous SNP variants identified in the FALCON assembly to generate phased contigs corresponding to the 2 alleles. The diploid nature of the genome is thereby captured in the assembly by a set of primary contigs with divergent haplotypes represented by a set of additional contigs called haplotigs. Genomic regions with low heterozygosity are represented as collapsed haplotypes in the primary contigs. Genome assemblies were run on an SGE-managed cluster using up to 30 nodes, where each node has 512 Gb of RAM distributed over 64 slots. The same configuration files were used for both species (Additional file 1). Three rounds of contig polishing were performed. For the first round, as part of the FALCON-Unzip pipeline, primary contigs and secondary haplotigs were polished using haplotype-phased reads and the Quiver consensus caller. For the second and third rounds of polishing, using the “resequencing” pipeline in SMRTLink v. 3.1, primary contigs and haplotigs were concatenated into a single reference, and BLASR (v. 3.1.0) was used to map all raw reads back to the assembly, followed by consensus calling with Arrow.

### Genome completeness

To assess quality and completeness of the assemblies, we used a set of 248 highly conserved eukaryotic genes from the CEGMA human set (CEGMA, RRID:SCR_015055) [23] and located them in each of the assemblies compared in this study. We used the human gene set because it is the phylogenetically closest set of birds available since all other CEGMA gene sets are from non-vertebrates. Briefly, the CEGMA human peptides were aligned to each genome using genblastA (command: genblast_v138_linux_x86_64 -p genblasta -t ${genome} -q ${CEGMA_genes} -c 0.3 -e 0.00001 -gff -pid -r 1, where ${genome} is the assembly and ${CEGMA_genes} is the CEGMA file; the output file contains the alignment percentage for each gene) [55]. The regions showing homology were then used to build gene models with exonerate [56], which were then assessed for frameshifts (command: exonerate -m protein2genome –percent 30 –bestn 1 –showtargetgff –ryo “>%qi\n%tcs\n%m\n” -q CEGMA_prot.fa -t contig.fa, where CEGMA_prot.fa is a CEGMA peptide and contig.fa is the corresponding contig in the assembly). In addition, we queried each genome for a set of 303 eukaryotic conserved single-copy genes, as well as 4915 conserved single-copy genes from 40 different avian species using the BUSCO v. 2.0 pipeline (BUSCO, RRID:SCR_015008) [26].

To compare protein amino acid sequence size between the CEGMA and BUSCO datasets, we performed blastp of each CEGMA sequence against the ancestral proteins of the target BUSCO dataset. We took the single best hit with an e-value cut off of 0.001 and extracted the CEGMA and BUSCO protein length values. We then ran a one-sided paired Wilcoxon signed-rank test of the 2 lengths for each protein (using the “wilcox.test” function with “paired = T.” in R).

### Gene prediction

Gene predictions for the zebra finch PacBio-based assembly were conducted by running Augustus gene prediction software (Augustus: Gene Prediction, RRID:SCR_008417; v. 3.2.2) [37] on the contigs and incorporating the Illumina short-read RNA-Seq brain data aligned with Tophat2 (TopHat, RRID:SCR_013035; v. 2.0.14) [28] as hints for possible gene structures. The data consisted of 146 126 838 paired-end reads with an average base quality score of 36. Augustus produces a distribution of possible gene models for a given locus, and models that are supported by our RNA-Seq data are given a “bonus” while the gene models not supported by RNA-Seq data are given a “penalty.” This results in the gene model most informed by biological data being selected as the most likely gene model for that locus.

We did not have Illumina transcriptome data for Anna's hummingbird, so standard Augustus gene prediction (v. 3.2.2) was used with both a chicken and human training background to determine the sequence predictions of the genes examined. The human-based predictions captured more of the divergent 5’ ends of the longer genes (*SLIT1* and *FOXP2*) than the chicken-based predictions, so a combination of both was used to produce the final sequences in this manuscript.

### RNA-Seq

RNA sequencing was centered around vocal learning brain regions in the zebra finch and will be described in more detail in a later publication. We utilized our data here for population analyses of assembly quality and for initial annotations. In brief, following modifications of a previously described protocol [30], 9 adult male zebra finches were kept isolated in soundproof chambers for 12 hours in the dark to obtain brain tissue from silent animals. Then brains were dissected from the skull and sectioned to 400 microns using a Stoelting tissue slicer (51 415). The sections were moved to a petri dish containing cold PBS with proteinase inhibitor cocktail (11 697 498 001). Under a dissecting microscope (Olympus MVX10), the 4 principle song nuclei (Area X, LMAN, HVC, and RA), as well as their immediate adjacent brain regions, were microdissected using 2-mm fine scissors and placed in microcentrifuge tubes. The samples were stored at –80°C. Then RNA was isolated and quantified, and samples of 2 birds were then pooled for each replicate, resulting in 5 replicates (1 single animal in one). RNA was converted to cDNA, library preparation was performed using the NEXTflex™ Directional RNA-Seq Kit (Illumina), and paired-end reads were sequenced on an Illumina HiSeq 2500 system. Adapters and poor-quality bases (<30) were trimmed using fastq-mcf from the ea-utilities package, and reads were aligned to assemblies using Tophat2 (v. 2.0.14).

### Chip-Seq

Three adult male zebra finches were treated as above, the brains were dissected, and the RA and surrounding arcopallium of each bird was then processed individually using the native ChIP protocol described in Brind’Amour et al. [57] with an H3K27ac antibody (Ab#4729). The DNA libraries were prepared using the MicroPlex Library Preparation Kit v. 2 (C05010012); 50-bp single-end sequencing was done on the Illumina HiSeq 4000 system. The reads were aligned to the assemblies using Bowtie2 (Bowtie, RRID:SCR_005476; v. 2.2.9) [31]. More detail will be provided in a later publication focusing on vocal learning brain regions.

### Comparative analyses between assemblies for individual genes

The Sanger-based reference zebra finch assembly in the UCSC browser, the Illumina-based reference Anna's Hummingbird in Avianbase [58], and both in NCBI were used for comparing with the PacBio assembly. In the UCSC browser, there are 2 annotations, 1 from 2008 [59] and the other from 2013 [60], with some differences between them. Our findings were similar, although not always identical, with both annotations, with errors being present in both annotations based on the PacBio assembly. The nucleotide quality score tract was only available in the 2008 browser.

Multiple species sequence alignments were done with BioEdit v. 7.2.5 [61, 62]. Dotplots of alignments were generated with Gepard v. 1.4 [63, 64]. Alignments of raw SMRT genome reads to the assembled genomes were done with Blasr, which is part of SMRTLink software from PacBio. Iso-Seq reads were aligned with GMAP (v. 2016–08-16) [65, 66].

## Availability of data

This Whole Genome Shotgun project has been deposited at DDBJ/ENA/GenBank under BioProject PRJNA368994. The zebra finch accession number is MUGN00000000, and SRA for raw reads is SRS1954332. The Anna's Hummingbird accession number is MUGM00000000, and SRA is SRP061272. The NCBI accessions also contain translation tables of the PacBio contig designations and their corresponding NCBI accession labels for all contigs (ftp://ftp.ncbi.nlm.nih.gov/genomes/all/GCA/002/008/985/GCA_002008985.2_Tgut_diploid_1.0/GCA_002008985.2_Tgut_diploid_1.0_assembly_report.txt and ftp://ftp.ncbi.nlm.nih.gov/genomes/all/GCA/002/021/895/GCA_002021895.1_Canna_diploid_1.0/GCA_002021895.1_Canna_diploid_1.0_assembly_report.txt, respectively; the first column contains the PacBio assembly contig ID, and the fifth column designates the corresponding NCBI contig accession number). We have also included these tables here as additional files (Tables S1 and S2). Supporting assemblies, BUSCO and CEGMA output files, and RNASeq and ChipSeq data are also available from the *GigaScience* respository, *Giga*DB [67].

## Additional Files

Supporting data are included in Supplementary Figs S1–S15 and Supplementary Tables S1–S2.

Supplementary Figure S1. DNA isolation, library construction, and size selection. (A) Pulsed-field gel showing original size of starting genomic DNA (lane 3), the sheared DNA (1), and the size-selected library (2). (B) Bioanalyzer trace before (blue) and after (red) library size selection for fragments >17 kb.

Supplementary Figure S2. Read and insert length distributions. (A, B) Sequence read length distributions from SMRT Cell sequencing for both species. (C, D) Sequenced DNA insert length distributions from SMRT Cell sequencing for both species.

Supplementary Figure S3. Box plots comparing protein coding sequence lengths of orthologous proteins between the CEGMA and BUSCO eukaryotic and avian datasets. **P < 0.001, ***P < 0.0001, one-sided paired Wilcoxon signed-rank test, prediction of the proteins being longer in CEGMA datasets.

Supplementary Figure S4. Vocal learning and adjacent brain regions in songbirds used for RNA-Seq and ChIP-Seq analyses, and comparison with humans. (A) Drawing of a zebra finch male brain section showing specialized vocal learning pathway and associated profiled song nuclei RA, HVC, LMAN, and Area X. (B) Drawing of a human brain section showing spoken-language pathway and analogous brain regions. Black arrows: posterior vocal motor pathway; white arrows: anterior vocal learning pathway; dashed arrows: connections between the 2 pathways; red arrow: specialized direct projection from forebrain to brainstem vocal motor neurons in vocal learners. Italicized letters adjacent to the song and speech regions indicate regions (in songbirds) that show mainly show motor (*m*), auditory (*A*), equally both motor and auditory (*m/a*) neural activity or activity-dependent gene expression. Figure from [68] and [4].

Supplementary Figure S5. Dot plot of sequence comparisons for genome assemblies of the *EGR1* region. (A) Comparison of zebra finch PacBio-based vs Sanger-based assemblies for the region containing *EGR1*, showing the GC-rich promoter region and closing and corrections of gaps for the PacBio-based assembly. (B) Comparison of hummingbird Illumina-based vs PacBio-based assemblies for the region containing *EGR1*, showing an erroneous tandem duplication in the Illumina-based assembly and closing of gaps for the PacBio-based assembly.

Supplementary Figure S6. Single SMRT genomic reads and Iso-Seq mRNA reads supporting PacBio *EGR1* assembly. (A) Zebra finch PacBio SMRT reads (rows) mapped against the zebra finch PacBio assembly (contig 405, entire *EGR1* region, same as Fig. 3A). Reads are shaded by length (>10 kb reads = black). (B) Example of a single ruby-throated hummingbird Iso-Seq read mapped against Illumina-based (top) and PacBio-based (bottom) Anna's hummingbird genome assemblies using GMAP. Note the first exon (blue) that is present in the Iso-Seq read is missing in the Illumina-based assembly, but present in the PacBio-based assembly.

Supplementary Figure S7. Dot plot of sequence comparison for the PacBio-based hummingbird and zebra finch *EGR1* region assemblies. Note regions of high species conservation and divergence surrounding *EGR1*. Blue box: location of the *EGR1* exons and intron.

Supplementary Figure S8. Dot plot comparisons for *DUSP1* region assemblies. (A) Comparison of the Sanger-based and PacBio-based zebra finch *DUSP1* region assemblies, showing problems in the Sanger-based assembly with microsatellite repeats. (B) Comparison of the Illumina-based and PacBio-based hummingbird *DUSP1* region assemblies, showing a large gap including the microsatellite region and the beginning of the gene, and an erroneous tandem duplication in the Illumina-based assembly.

Supplementary Figure S9. PacBio correction of base call errors found in the Sanger reference. (A) Confirmation that the Pacbio-based genome assemnly in the 3 locations is correct and different from the zebra finch reference is made by alignments with DUSP1 sequences of other songbird species. (B) PacBio reads (rows) corresponding to the genomic region in DUSP1 that differs in the 3 locations from the zebra finch Sanger reference, resulting in a.a. changes. The codons in question are highlighted.

Supplementary Figure S10. Dot plot comparison of assemblies for the *DUSP1* microsatellite region. (A) Differences in the microsatellite region upstream of the *DUSP1* protein coding sequence between the primary and secondary haplotypes in the fully assembled zebra finch PacBio-based assembly. (B) Differences in microsatellites region upstream of *DUSP1* between the zebra finch and hummingbird in the fully assembled PacBio-based assemblies.

Supplementary Figure S11. Dot plot comparisons for PacBio-based *DUSP1* region assemblies with orthogonal validation. Comparison of the PacBio-based genome assembly and Sanger-based single clone of the (A) zebra finch and (B) hummingbird *DUSP1* upstream region assemblies showing more consistency between the 2 (than in Fig S8A). Not visible in this high-level alignment view is an 11-bp deletion and several SNPs in this allele of the PacBio contig relative to the other allele; the single clone of the individual is more consistent with the alternate allele without the 11-bp deletion.

Supplementary Figure S12. Single Iso-Seq mRNA reads supporting PacBio assemblies. (A) Full-length PacBio mRNA sequence Iso-Seq ruby throated hummingbird reads for DUSP1 aligned against the exons of the corresponding primary contigs from Anna's hummingbird Illumina (top panel) and PacBio (bottom panel) assemblies. (B) Similar alignments for FOXP2 IsoSeq reads.

Supplementary Figure S13. Dot plot comparison of assemblies for the *FOXP2* region. (A) Zebra finch. (B) Hummingbird.

Supplementary Figure S14. (A) Multiple sequence alignment of the FOXP2 protein for the 4 assemblies (2 zebra finch and 2 hummingbird) compared in this study, showing correction of a nucleotide error in the Sanger-based zebra finch assembly and correction of an erroneous stop codon (x) in the Illumina-based hummingbird assembly. Note an extra 18 a.a. stretch in the hummingbird sequence validated by gene prediction of both assemblies that was not present in the zebra finch. (B) Missing 88 bp of sequence in exon 6 of Illumina-based assembly. (C) Resolution of exon 6 in PacBio-based assembly, also revealing an SNP.

Supplementary Figure S15. Large regional correction made by the PacBio diploid assembly. (A) Correction of an erroneous stretch of 462 bp in the first intron of *FOXP2* in the hummingbird Illumina assembly by the PacBio assembly. (B) Dot plot of haplotype variation in the *FOXP2* gene revealed by the PacBio diploid assembly: a 708-bp deletion in the secondary haplotype contig relative to the primary contig.

Supplementary Figure S16. Dot plot comparison of assemblies for the *SLIT1* region. (A) Zebra finch. (B) Hummingbird.

## Abbreviations

A1-L4: primary auditory cortex—layer 4; aDLM: anterior dorsolateral nucleus of the thalamus; Am: nucleus ambiguous; Area X: a vocal nucleus in the striatum; aSt: anterior striatum vocal region; aT: anterior thalamus speech area; Av: avalanche; DM: dorsal medial nucleus of the midbrain; HVC: a vocal nucleus (no abbreviation); L2: auditory area similar to human cortex layer 4; LMC: laryngeal motor cortex; LSC: laryngeal somatosensory cortex; MAN: magnocellular nucleus of the anterior nidopallium; MO: oval nucleus of the anterior mesopallium; NIf: interfacial nucleus of the nidopallium; PAG: peri-aqueductal gray; RA: robust nucleus of the arcopallium; v: ventricle space.

## Competing interest

Jonas Korlach, Sarah Kingan, and Chen-Shan Chin are full-time employees at Pacific Biosciences, a company developing single-molecule sequencing technologies.

## Funding

This work was supported by HHMI funds to E.D.J. and PacBio funds to J.K.

## Author contributions

J.K. and E.D.J. designed the project and wrote the manuscript; C.S.C. and S.K. carried out genome assemblies; J.K., G.G., and S.K. conducted analyses on single genes as well as CEGMA and BUSCO analyses; G.G. and J.N.A. conducted RNA-Seq experiments, L.C. conducted Chip-Seq experiments; J.H. processed samples; and all authors contributed to writing and editing the manuscript.

## Supplementary Material

GIGA-D-17-00046_Original-Submission.pdf

GIGA-D-17-00046_Revision-1.pdf

Response-to-Reviewer-Comments_Original-Submission.pdf

Reviewer-1-Report-(Original-Submission).pdf

Reviewer-2-Report-(Original-Submission).pdf

Additional Files
