# Supplementary material for: De novo PacBio long-read and phased avian genome assemblies correct and add to reference genes generated with intermediate and short reads
Source: Gigascience. 2017 Aug 28;6(10):1–16. doi: 10.1093/gigascience/gix085 (PMC5632298; doi:10.1093/gigascience/gix085)
Supplement: Additional Files [file gix085_Supp.zip › Korlach et al supplementary figures v15.pdf]

A. Pulse-field gel of hummingbird genomic DNA

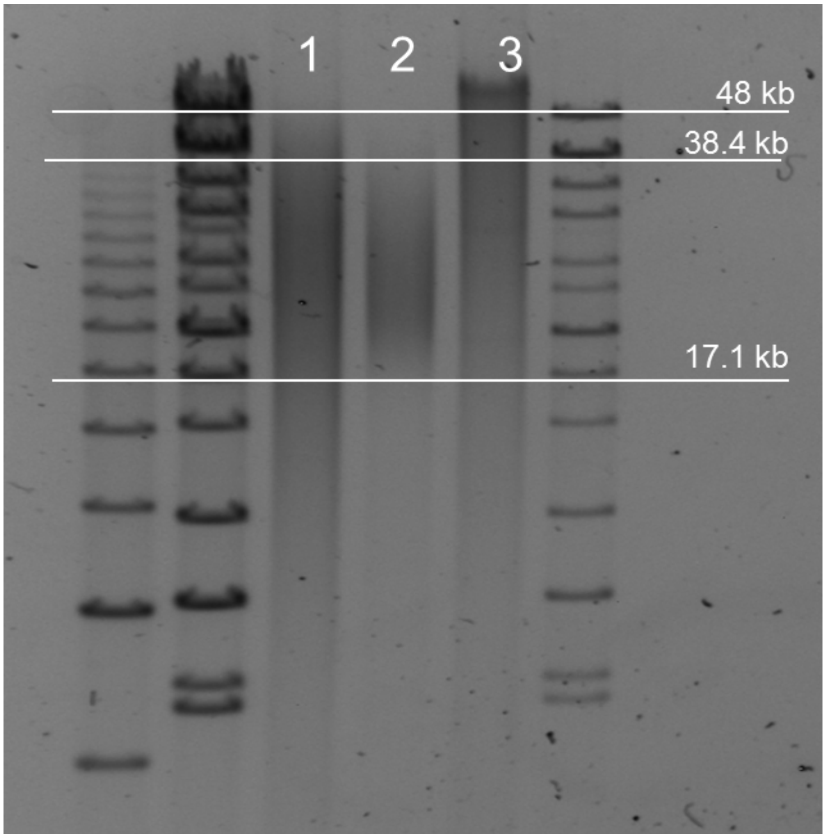

1: Sheared gDNA: 35 kb & 40 kb

2: BluePippin size-selected library (17 kb cut-off)

3: Original gDNA

B. Bioanalyzer plot of hummingbird SMRTbell library

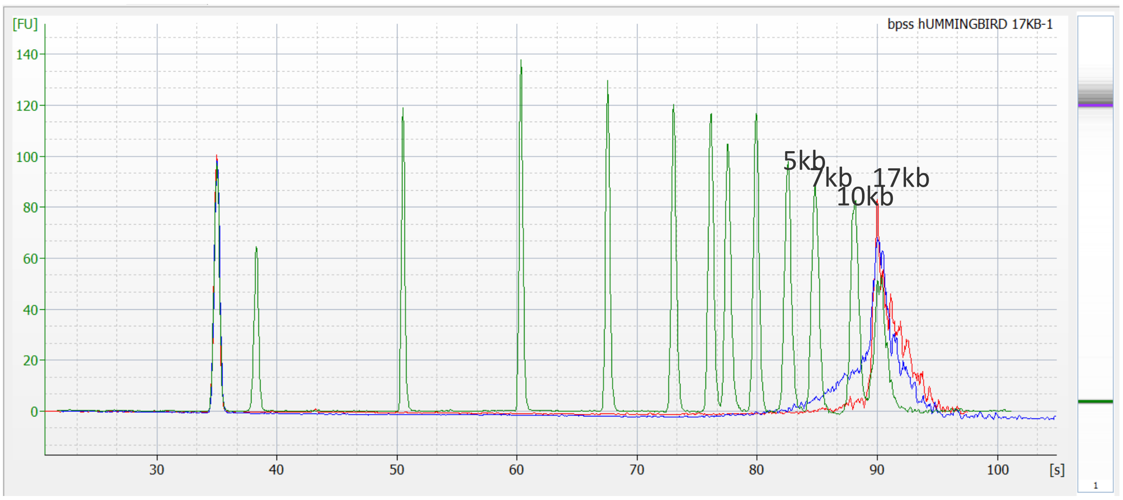

- Blue: SMRTbell library before size selection
- Red: SMRTbell library after Blue Pippin size selection (17kb cutoff)
- Green: Control DNA ladder

Figure S1

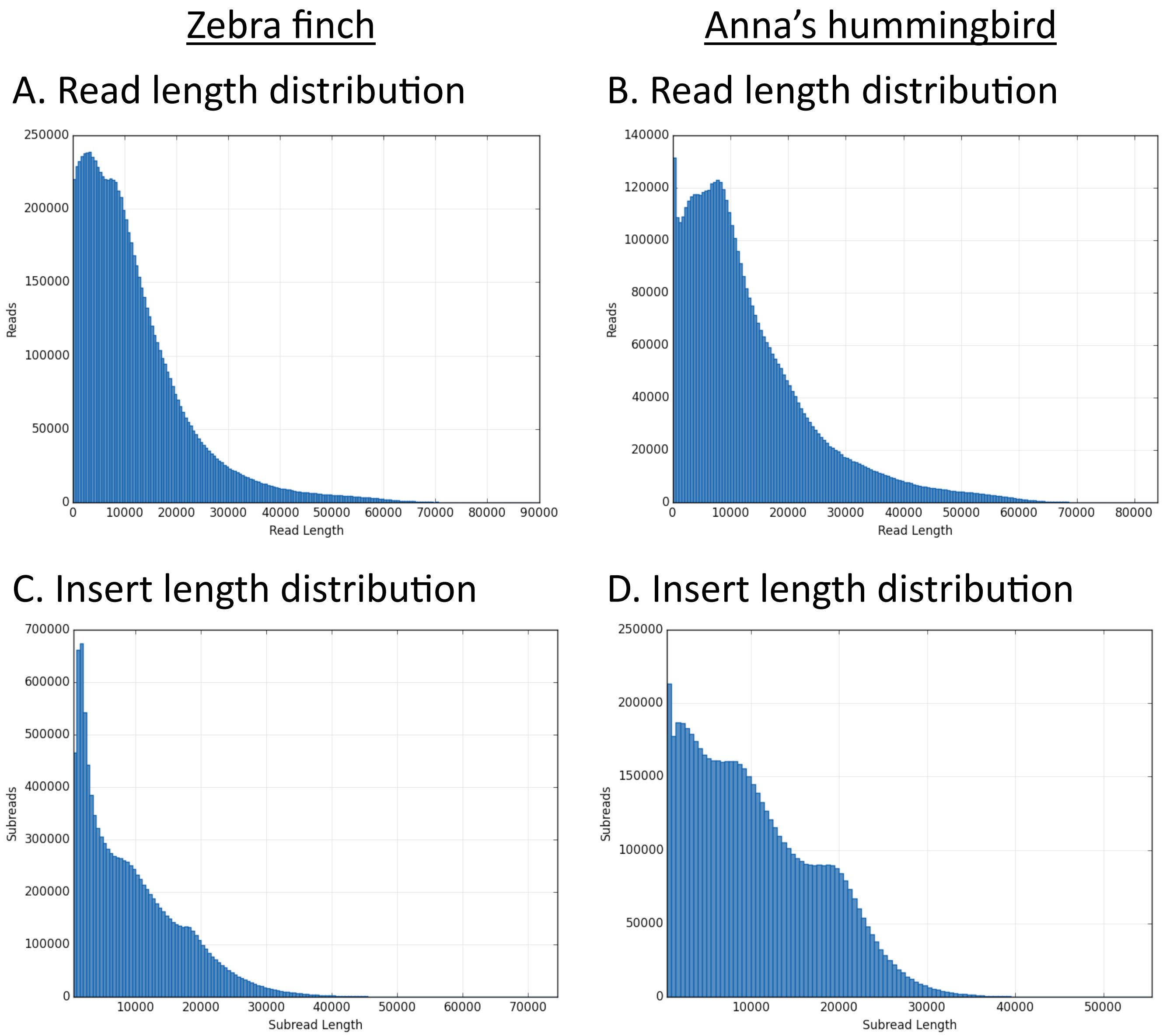

Zebra finch
Anna's hummingbird
A. Read length distribution
Reads
Read Length
B. Read length distribution
Reads
Read Length
C. Insert length distribution
Subreads
Subread Length
D. Insert length distribution
Subreads
Subread Length

Figure S2

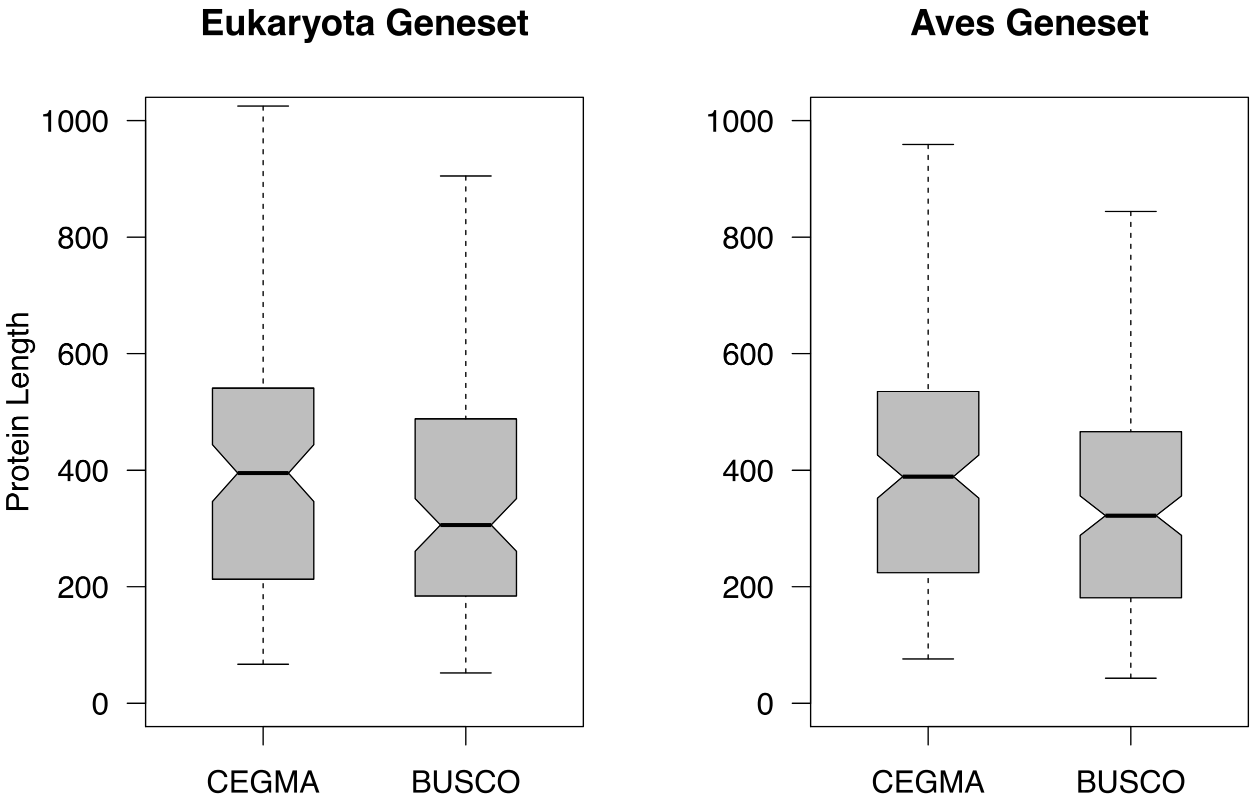

N = 144, W = 5683, $P < 0.001$

N = 177, W = 13376, $P < 0.001$

Figure S3

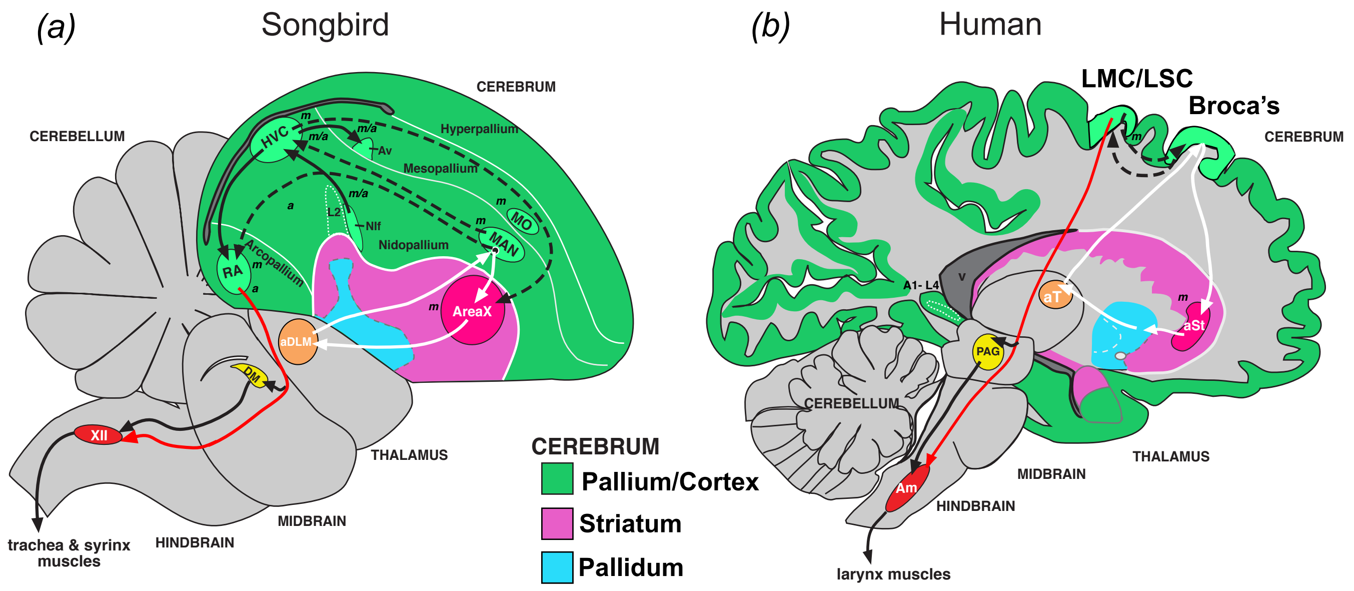

(a)
Songbird
CEREBRUM
CEREBELLUM
HVC
Hyperpallium
Av
Mesopallium
MO
L2
NIf
Nidopallium
MAN
RA
Arcopallium
AreaX
aDLM
DM
XII
THALAMUS
MIDBRAIN
HINDBRAIN
trachea & syrinx muscles
(b)
Human
LMC/LSC
Broca's
CEREBRUM
A1- L4
V
aT
aSt
PAG
CEREBELLUM
THALAMUS
MIDBRAIN
HINDBRAIN
Am
larynx muscles
CEREBRUM
Pallium/Cortex
Striatum
Pallidum

Figure S4

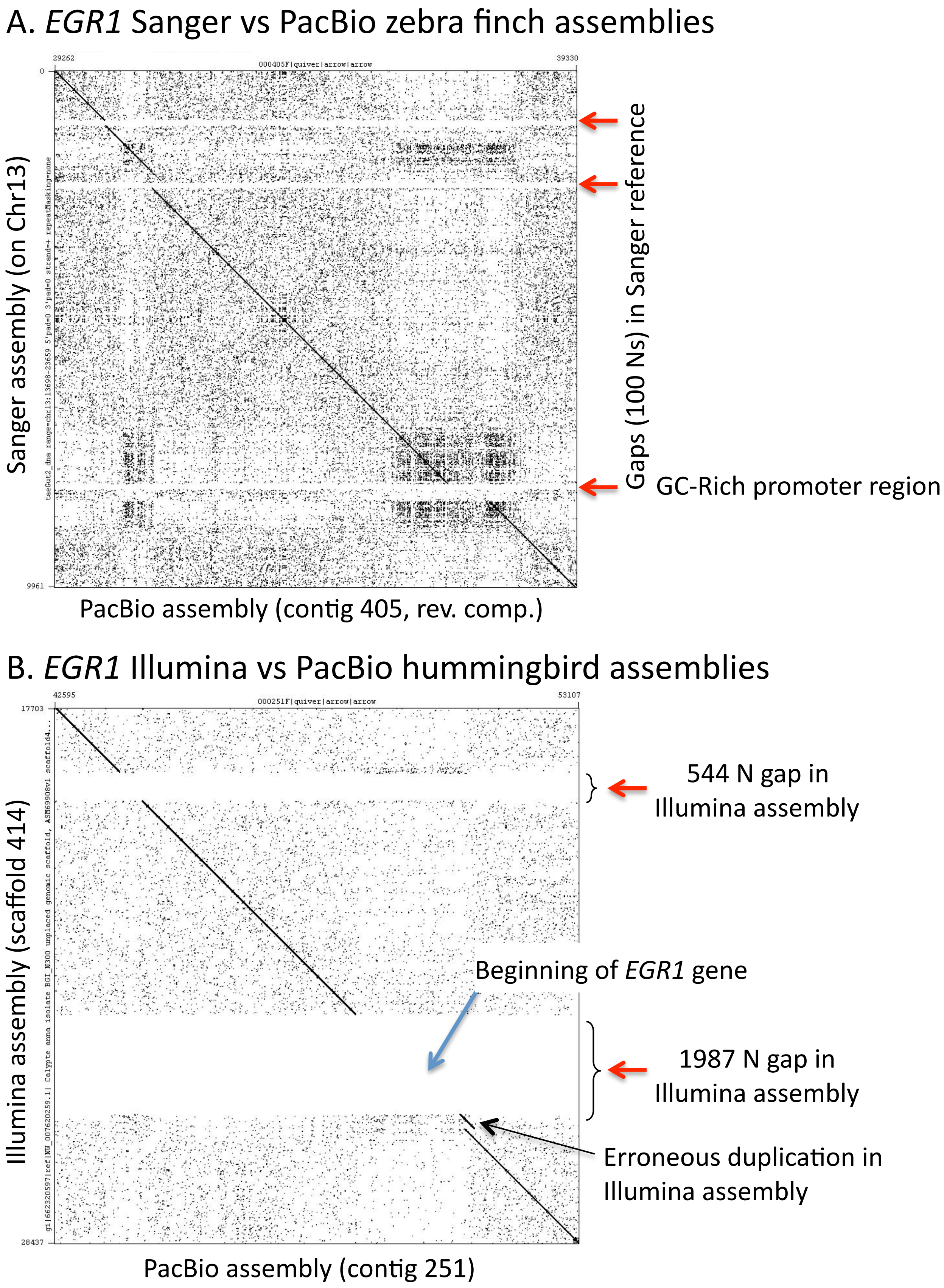

A. EGR1 Sanger vs PacBio zebra finch assemblies
29262
000405F|quiver|arrow|arrow
39330
0
9961
Sanger assembly (on Chr13)
Gaps (100 Ns) in Sanger reference
GC-Rich promoter region
PacBio assembly (contig 405, rev. comp.)
B. EGR1 Illumina vs PacBio hummingbird assemblies
42595
000251F|quiver|arrow|arrow
53107
17703
28437
Illumina assembly (scaffold 414)
544 N gap in
Illumina assembly
Beginning of EGR1 gene
1987 N gap in
Illumina assembly
Erroneous duplication in
Illumina assembly
PacBio assembly (contig 251)

Figure S5

A. Raw PacBio SMRT genome reads against *EGR1* Pacbio assembly

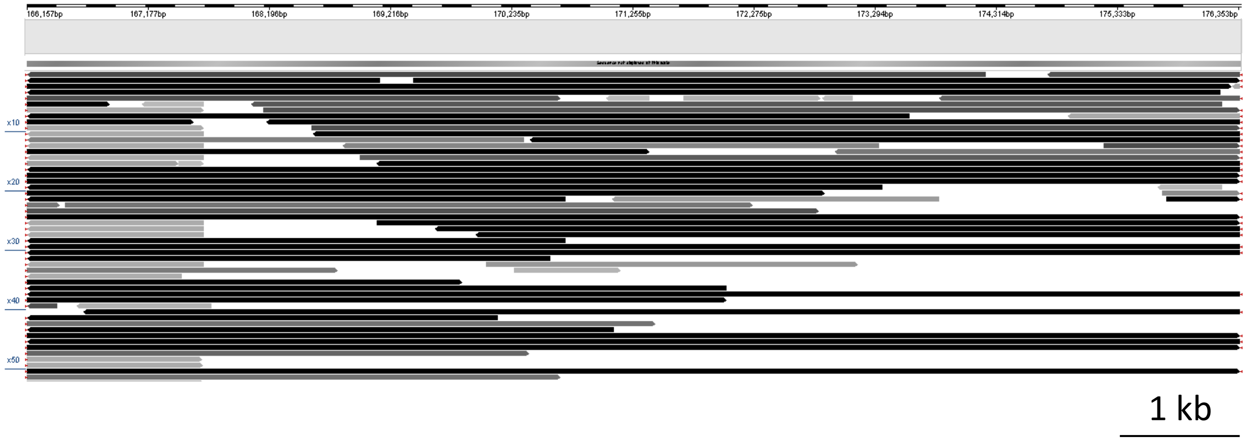

B. Raw PacBio IsoSeq mRNA read against *EGR1* assemblies

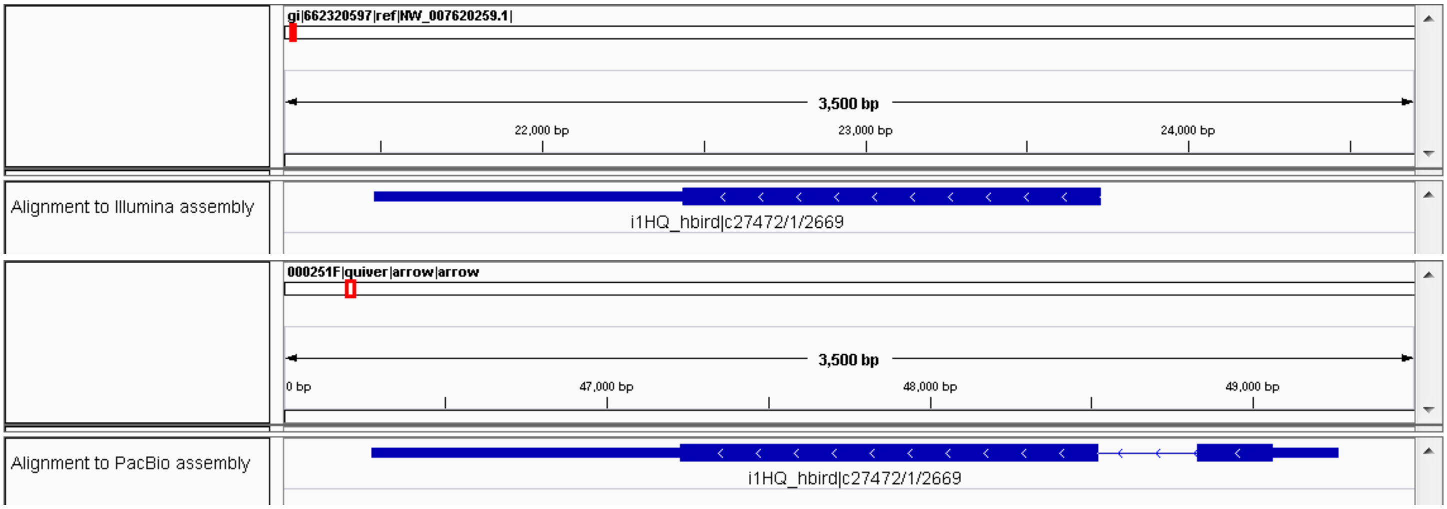

Figure S6

Hummingbird vs zebra finch *EGR1* region

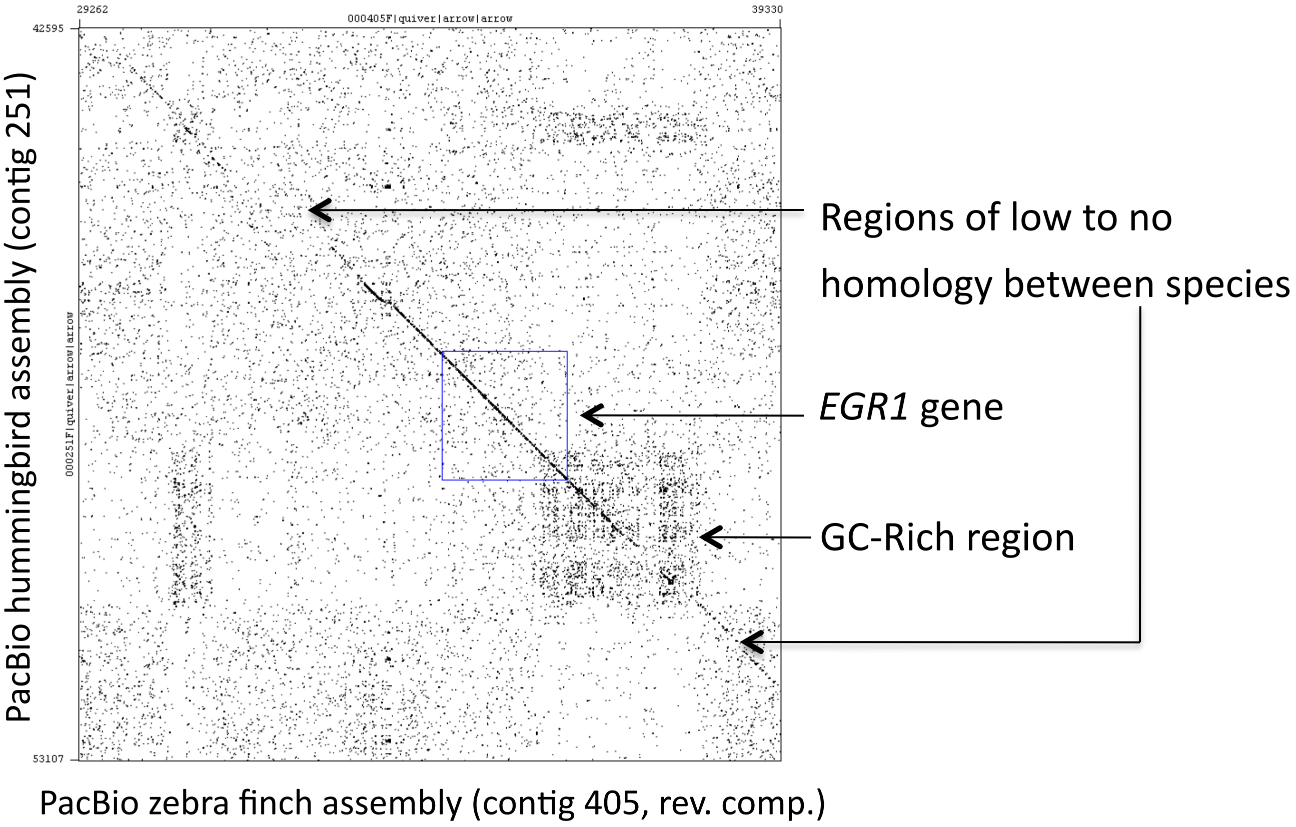

Figure S7

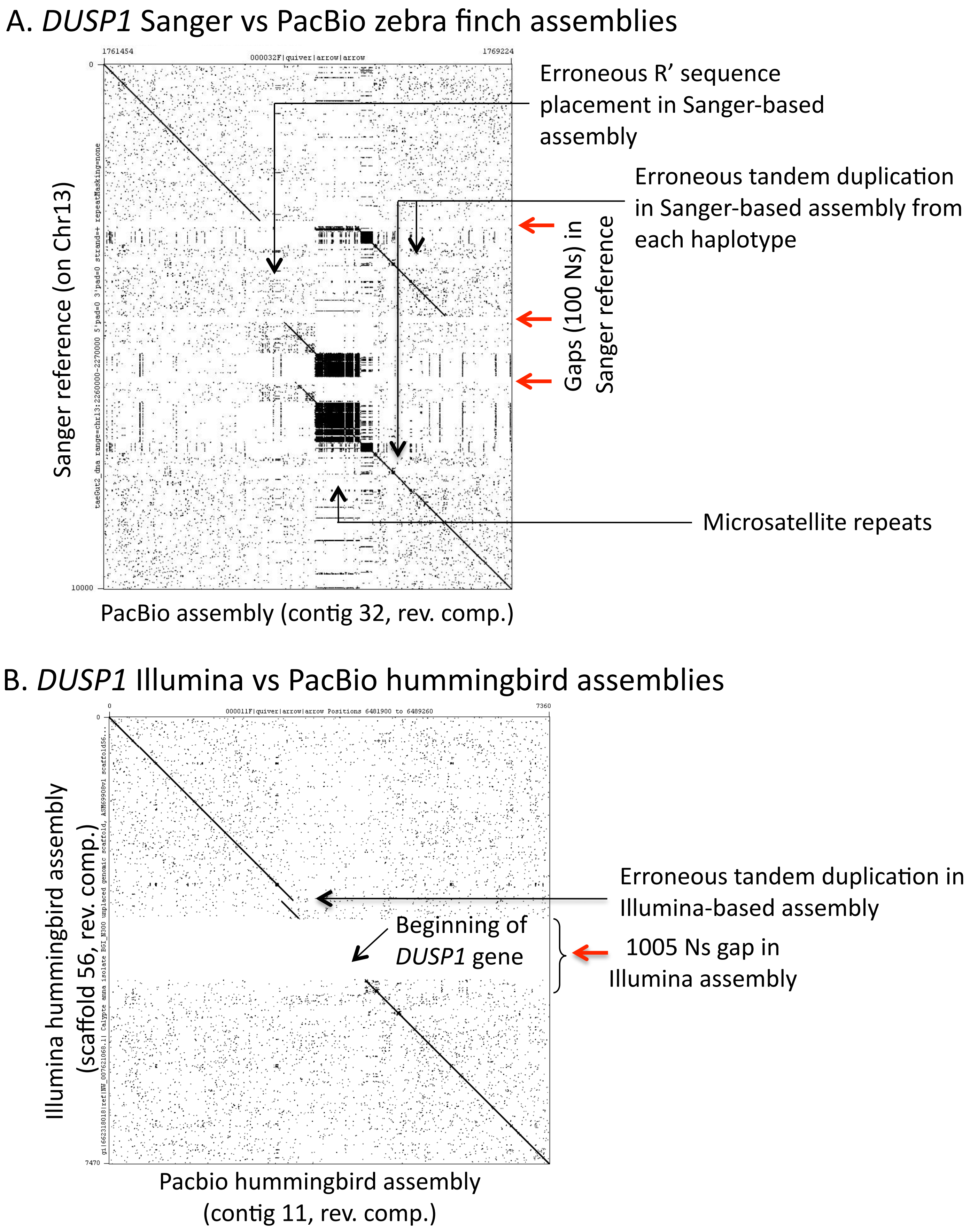

A. DUSP1 Sanger vs PacBio zebra finch assemblies
1761454
000032F|quiver|arrow|arrow
1769224
0
10000
Sanger reference (on Chr13)
Erroneous R’ sequence placement in Sanger-based assembly
Erroneous tandem duplication in Sanger-based assembly from each haplotype
Gaps (100 Ns) in Sanger reference
Microsatellite repeats
PacBio assembly (contig 32, rev. comp.)
B. DUSP1 Illumina vs PacBio hummingbird assemblies
0
000011F|quiver|arrow|arrow Positions 6481900 to 6489260
7360
0
7470
Illumina hummingbird assembly (scaffold 56, rev. comp.)
Erroneous tandem duplication in Illumina-based assembly
Beginning of DUSP1 gene
1005 Ns gap in Illumina assembly
Pacbio hummingbird assembly (contig 11, rev. comp.)

Figure S8

A. Apparent a.a. errors due to base call errors in Sanger finch reference

```
zebra_finch         QGLLHTVVLLDYRSADLEVPQRDSSMLFTLRLQFWHKN----
b_crowned_manakin   QGLFHTVVLLDERSADLDAPKRDSTMLLALGTLCREARGARI
c_flycatcher        QGLFHSVVLLDERSADLEAPKRDSTVLLALGTLCREARGARI
white_t_sparrow     QGLFHTVVLLDERSADLEMPKRDSTMLLALGTLCREARGARI
starling            QGLFHTVVLLDERSADLEVPKRDSTMLLALGTLCREARGARI
Great_tit           QGLFHTVVLLDERSADLEVPKRDSTMLLALGTLCREARGARI
ground_tit          QGLFHTVVLLDERSADLEVPKRDSTMLLALGTLCREARGARI
                    ***:*:***** *****: *:***::*::*      . .
```

B. Pacbio zebra finch *DUSP1* reads has same base calls as other species

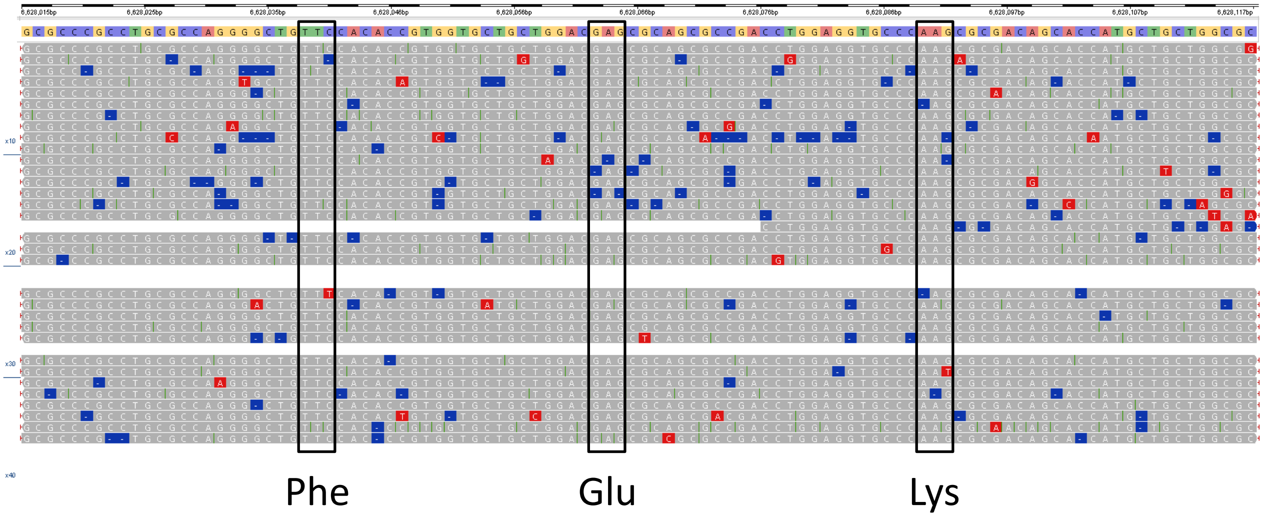

Figure S9

A. Haplotype differences in *DUSP1* microsatellite repeats

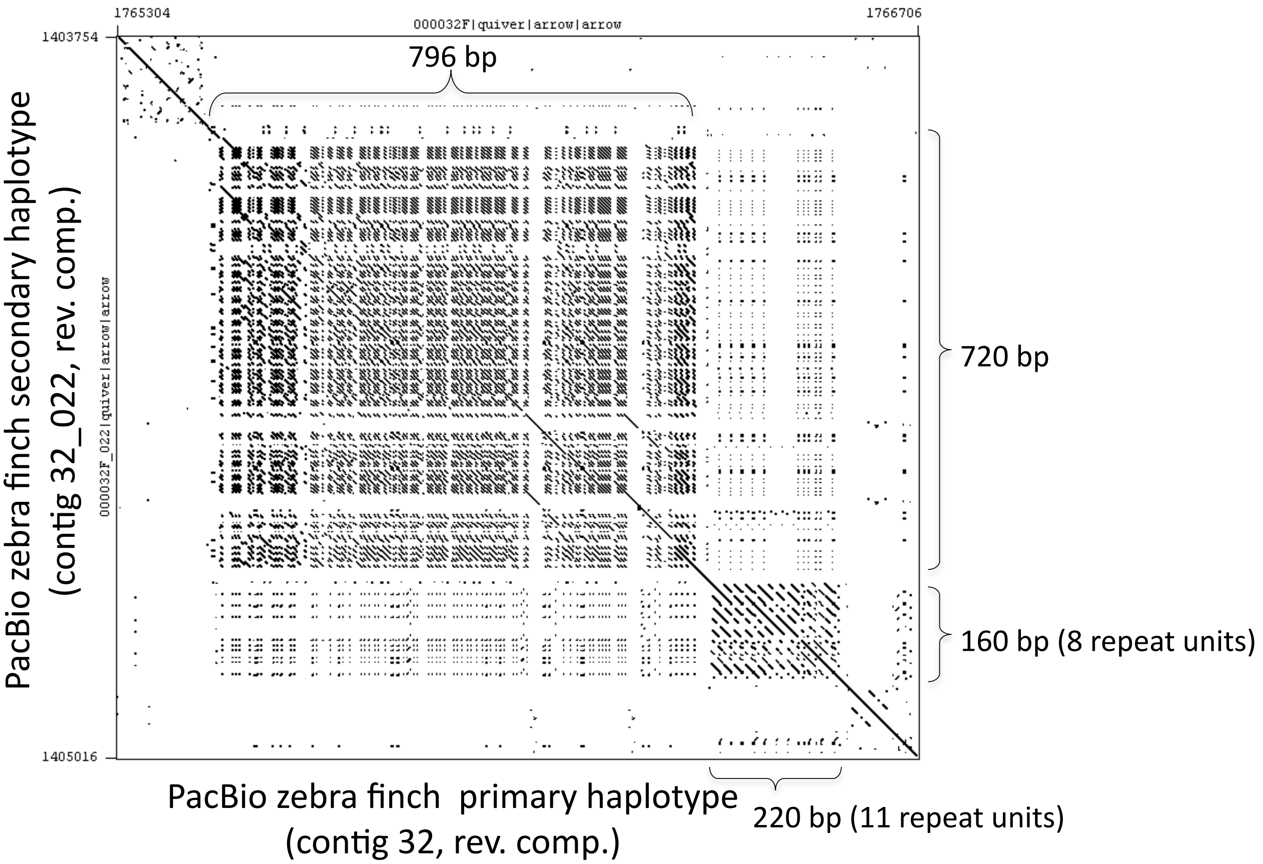

B. Species differences in *DUSP1* microsatellite repeats

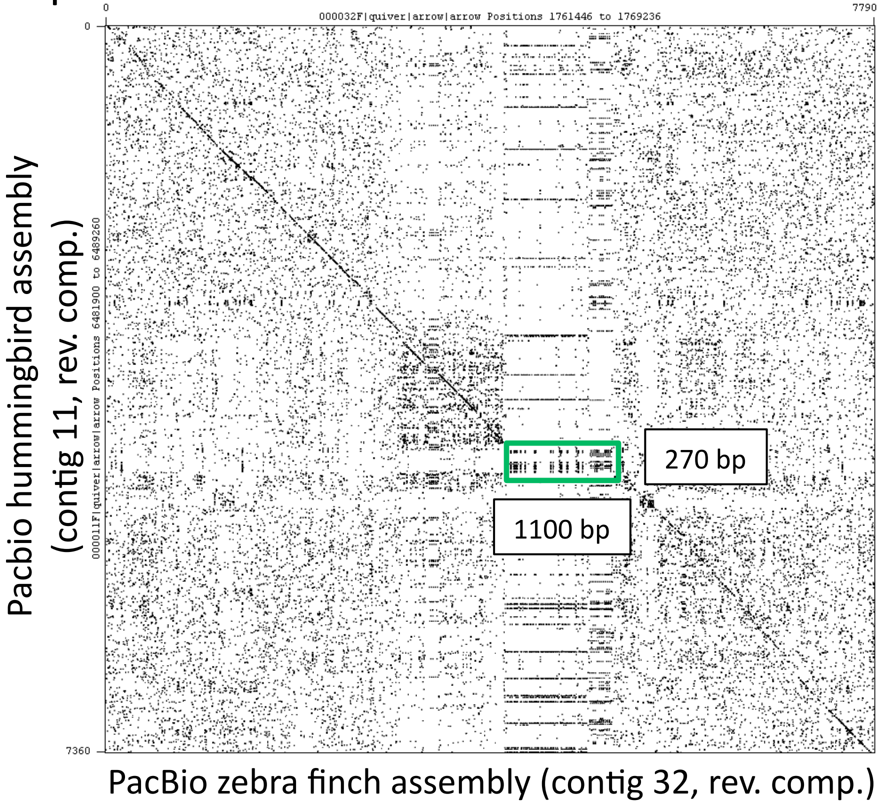

Figure S10

A. *DUSP1* PacBio vs single clone Sanger-based zebra finch assemblies

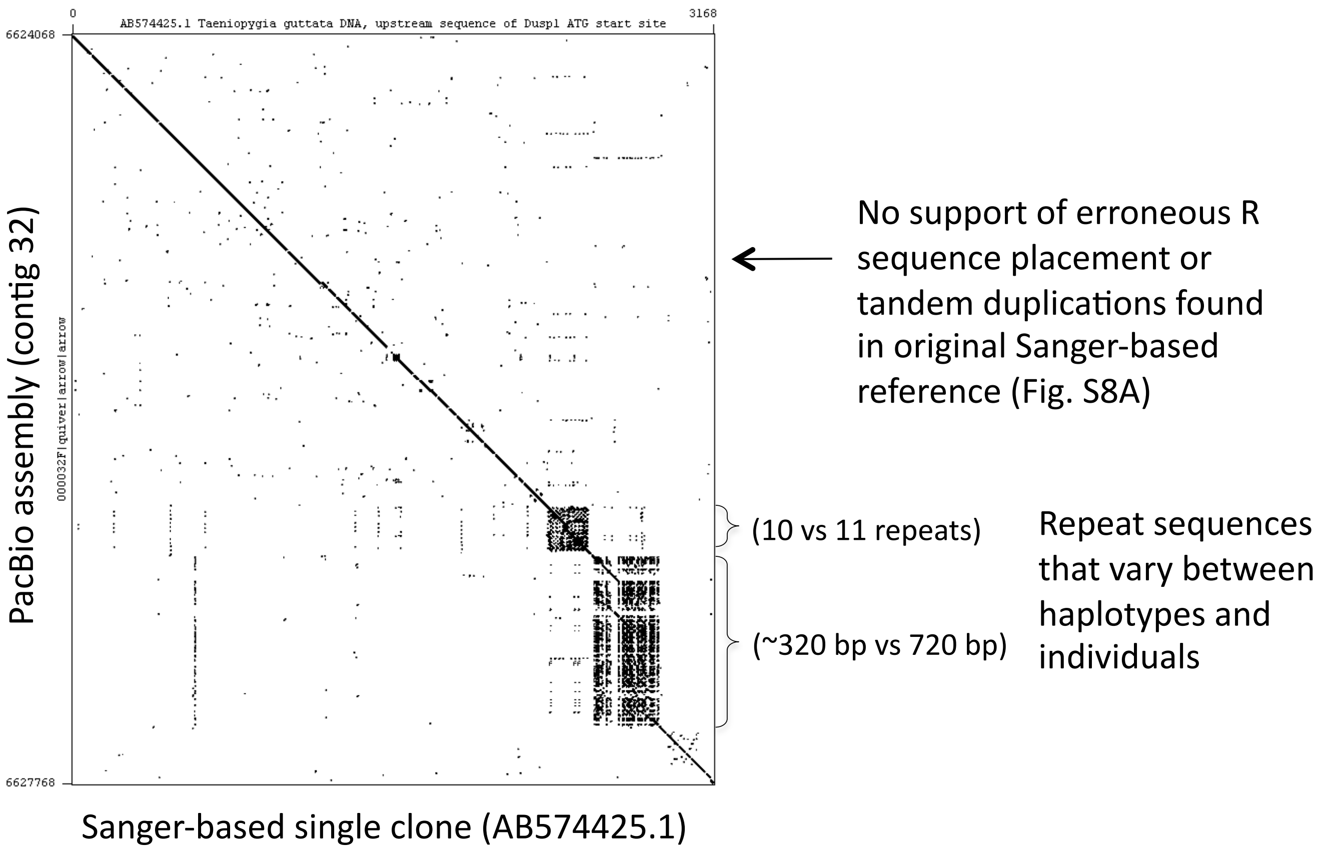

B. *DUSP1* PacBio vs single clone Sanger-based hummingbird assemblies

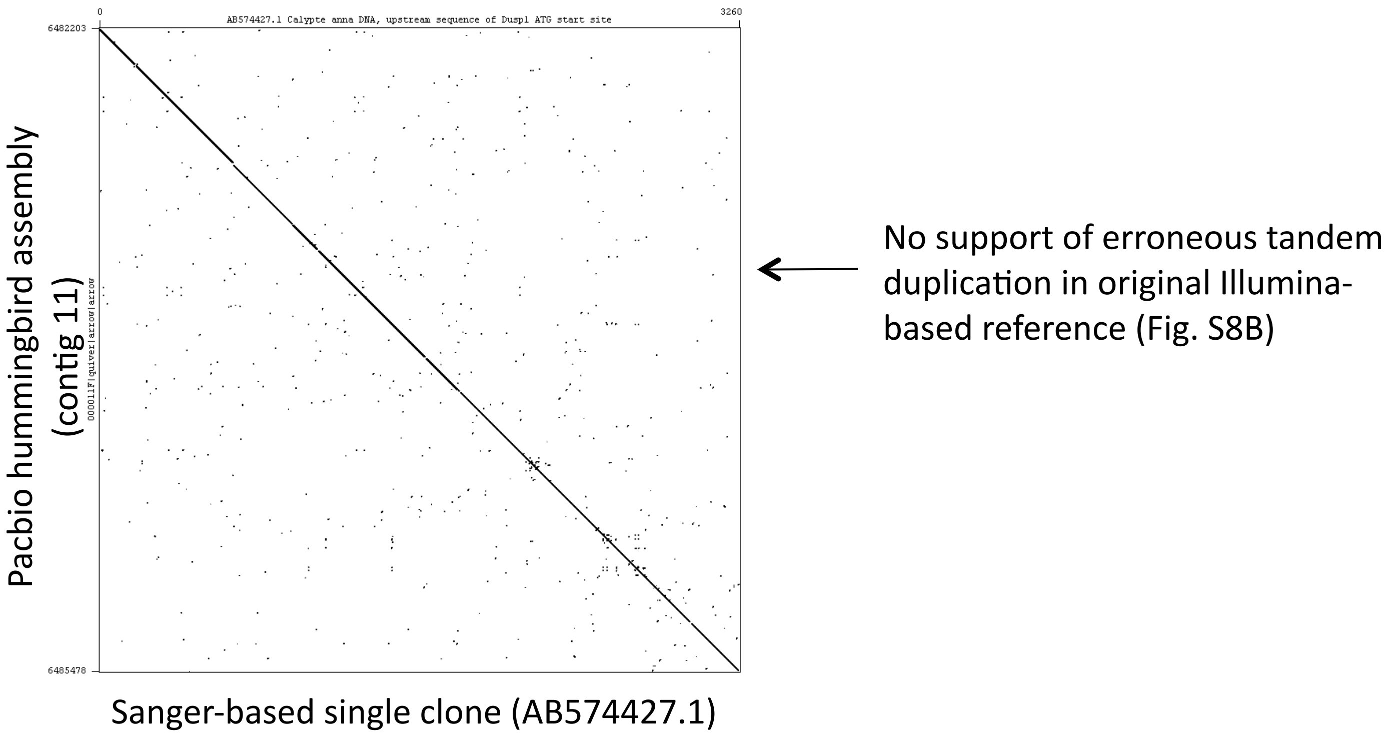

Figure S11

A. DUSP1

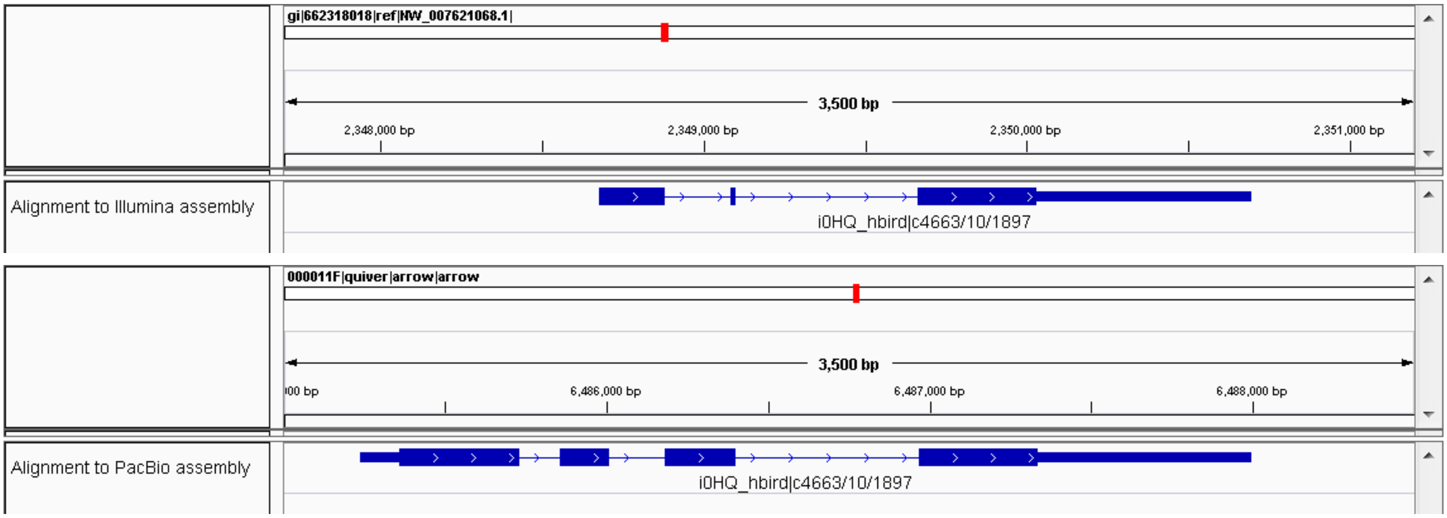

B. FOXP2

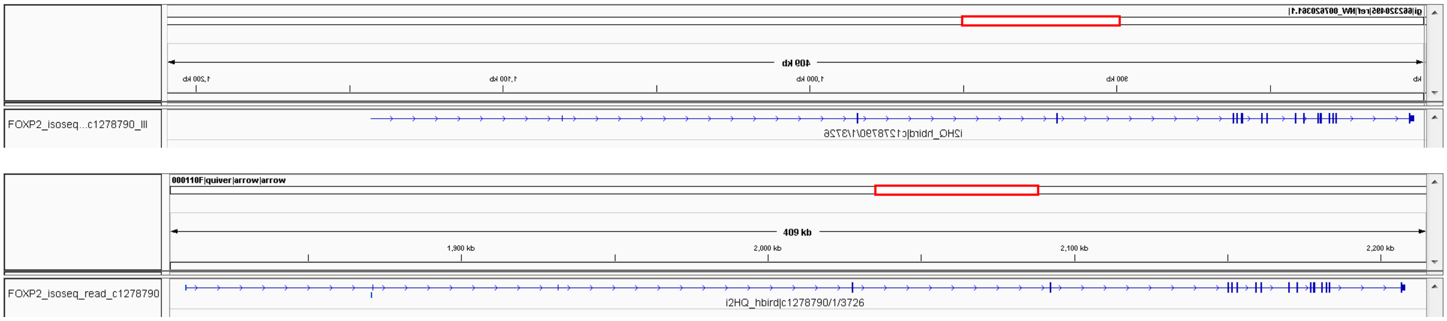

Figure S12

A. *FOXP2* Sanger vs PacBio zebra finch assemblies

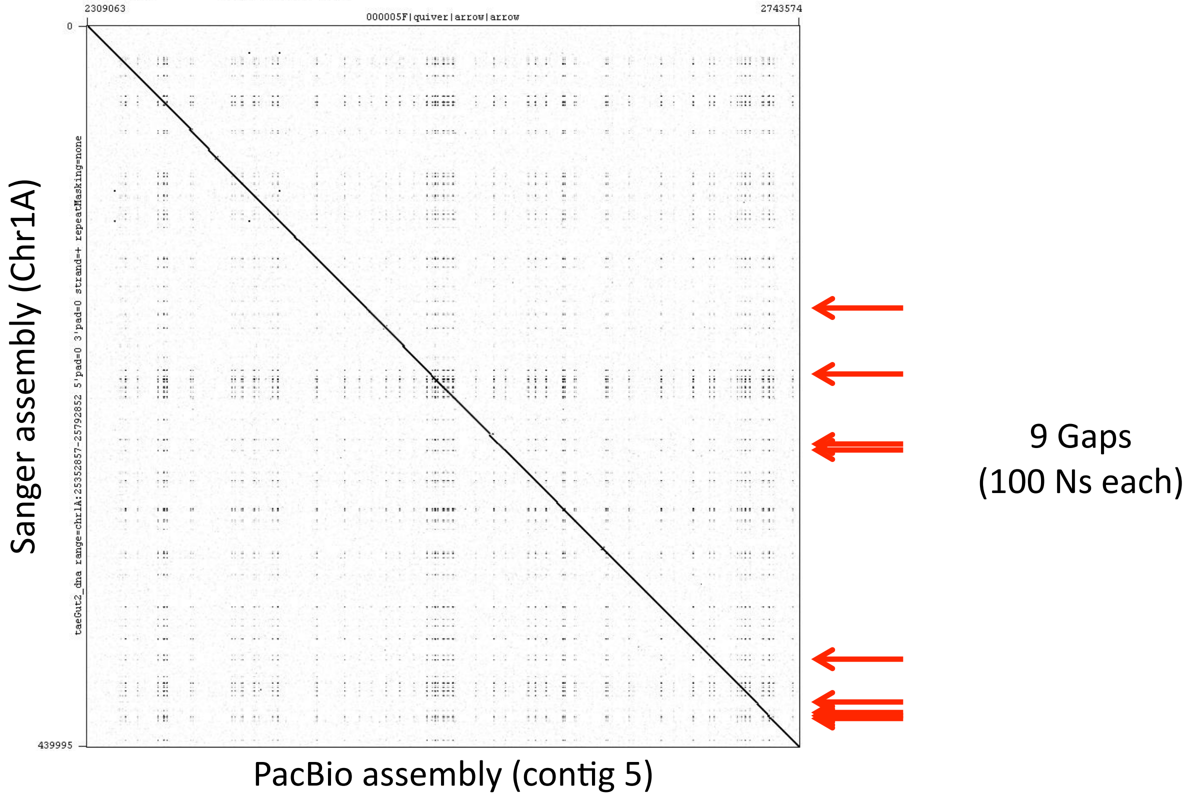

B. *FOXP2* Illumina vs PacBio hummingbird assemblies

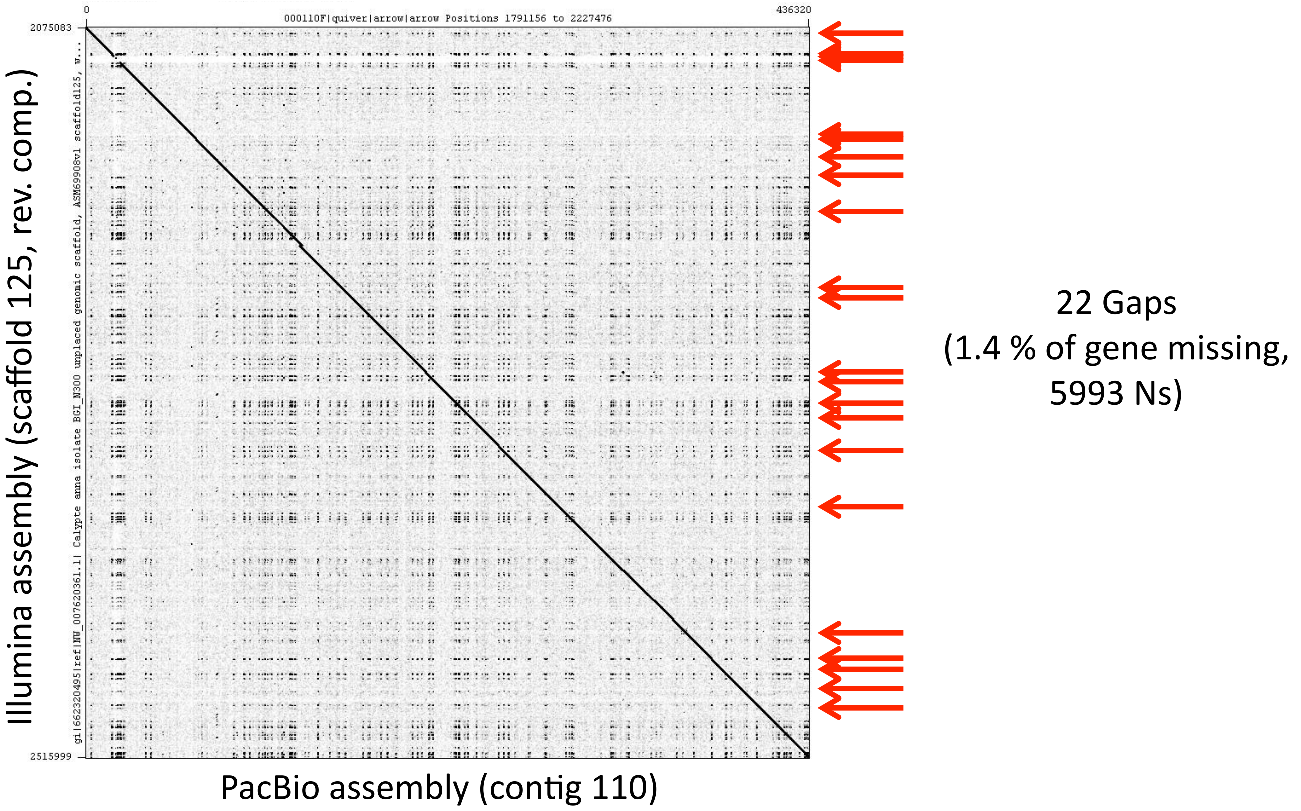

Figure S13

A. Predicted *FOXP2* protein alignment across assemblies and species

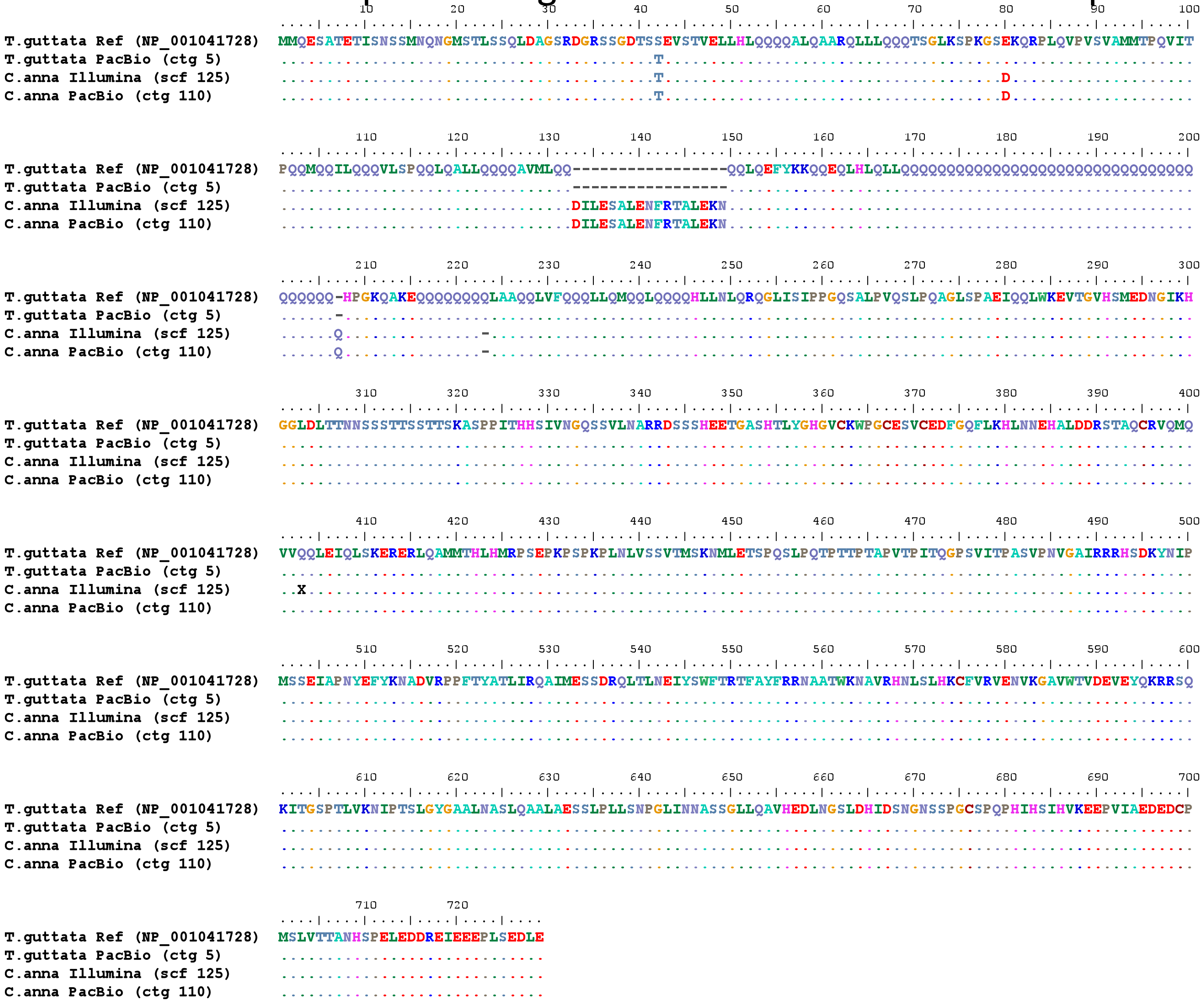

B. 88 Ns in the middle of exon 6 in Illumina assembly, making 2 exons

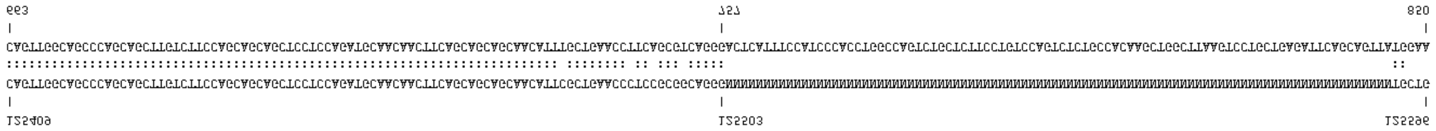

C. 1 contiguous exon, PacBio assembly (1 SNP relative to mRNA)

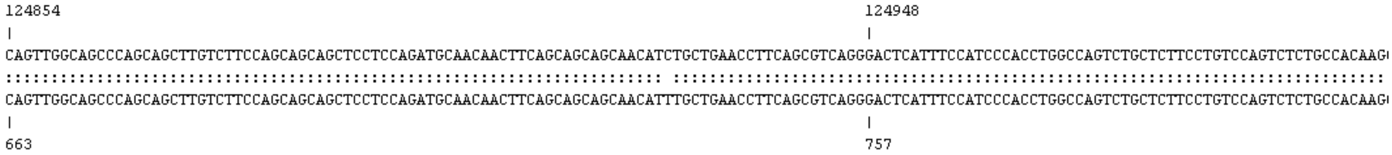

Figure S14

A. Correction of homonucleotide and large segment in *FOXP2* locus

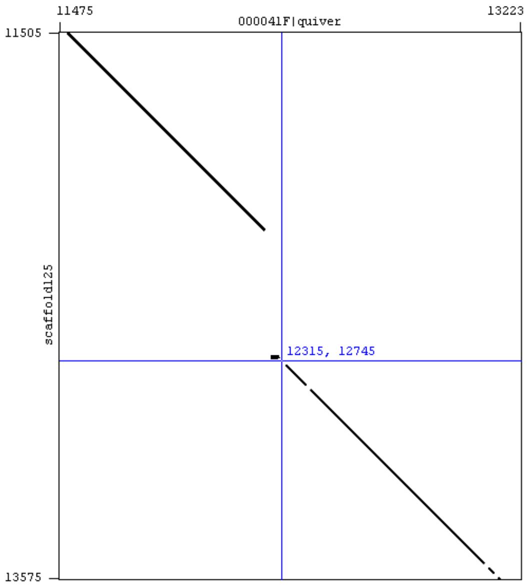

- Region in 1st intron:
- Illumina scaffold has 462 bp of additional sequence adjacent to the long T homonucleotide stretch.
- No read support for this stretch of sequence in the PacBio data

```
Position: 11475, 11505
12221                                                                                         12315                                                                                    12408
|                                                                                             |                                                                                            |
TTGGGGTTTTTTTTTAACCTAGAAGTTGTTTGTTGCAGGTGGTTTTATATTTTGGGCTTTTTTTTTTTTTTTTTTTTTTTTTTTTTTTTTTTTTTTTTTTTTTTTTTTCCTTGGCCTACAGAATGTTTTGTGAAAAAGAAACAGTTCATCTTTTCATGGTCTCTGAGGCTTTGTTATCATTTTCCTCGGGAGAAT
:  :   ::: : ::  ::::       ::        :     :     ::     : ::::: :::: ::::: ::::::::::::::::::::::::: :::::: : :::::::::::::::::::::::::::::::::::::::::::::::::::::::::::::::::::::::::::::
TAAGTAGTTTCTCTTTTCCTACTTAAGATTCCGCTGTTGATACATCCATGTTCACATTCTTTTTGTTTTCTTTTTCTTTTTTTTTTTTTTTTTTTTTTTTTTCTTGGCTTTCAGAATGTTTTGTGAAAAAGAAACAGTTCATCTTTTCATGGTCTCTGAGGCTTTGTTATCATTTTCCTCGGGAGAAT
|                                                                                             |                                                                                            |
12651                                                                                         12745                                                                                    12838
```

B. Large deletion in one haplotype of *FOXP2* locus

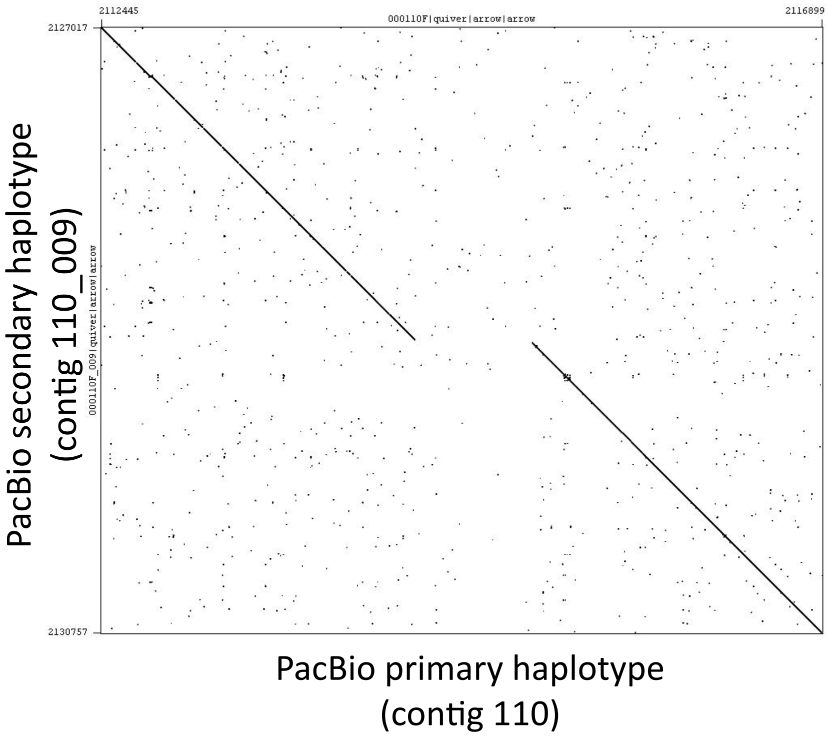

Figure S15

A. *SLIT1* Sanger vs PacBio zebra finch assemblies

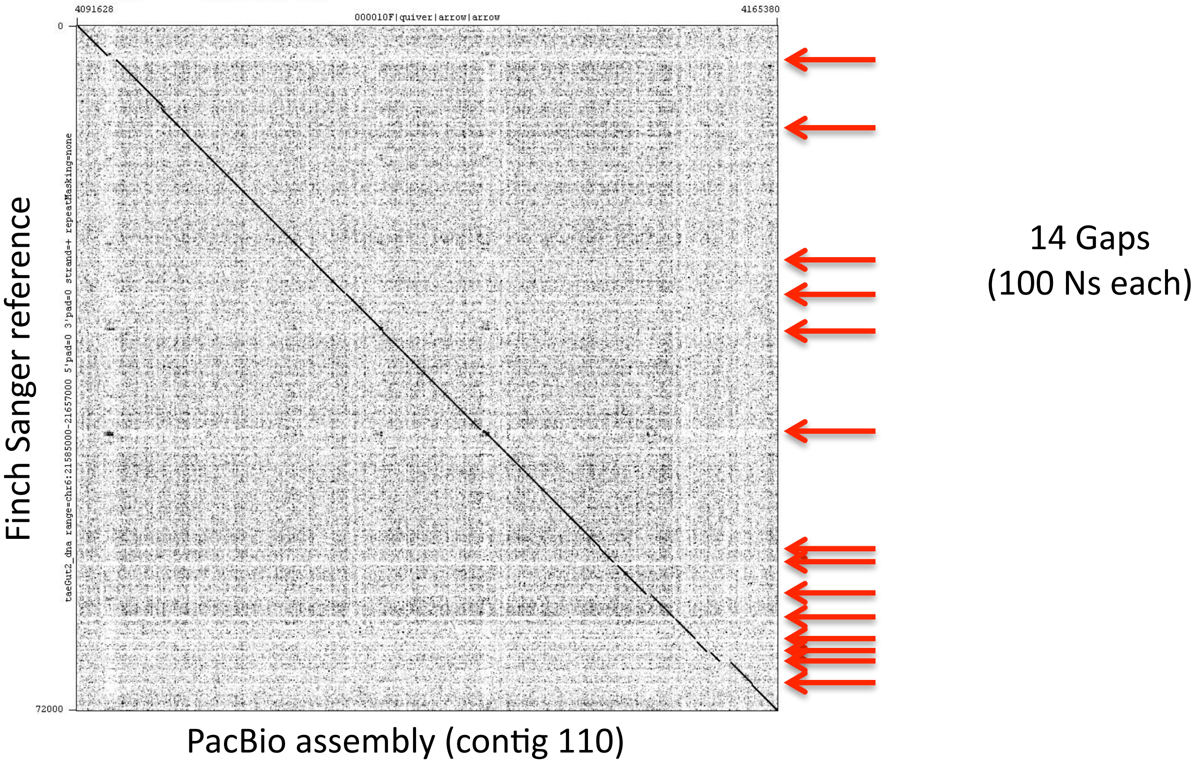

B. *SLIT1* Illumina vs PacBio hummingbird assemblies

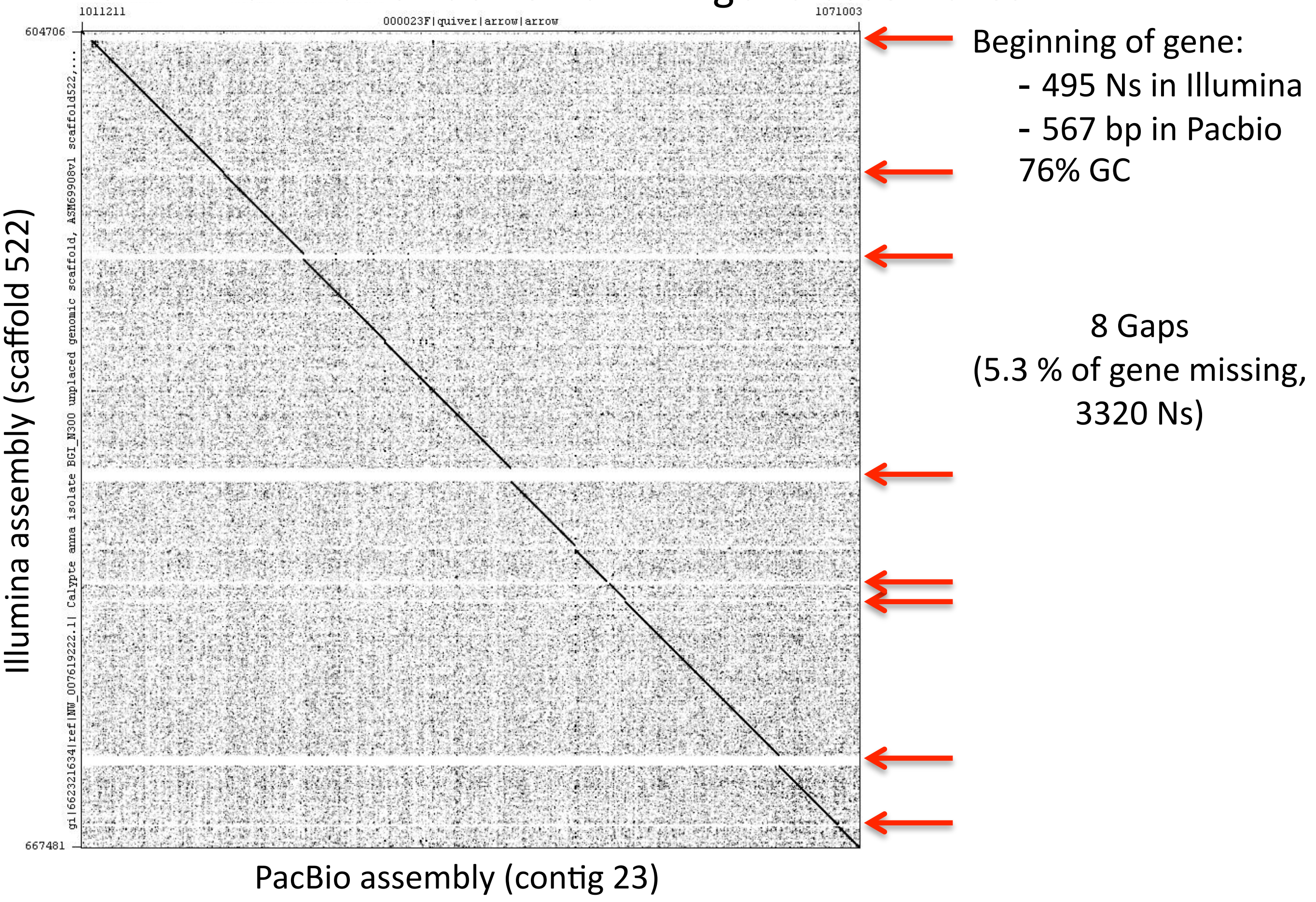

Figure S16
