# Supplementary material for: De novo PacBio long-read and phased avian genome assemblies correct and add to reference genes generated with intermediate and short reads
Source: Gigascience. 2017 Aug 28;6(10):1–16. doi: 10.1093/gigascience/gix085 (PMC5632298; doi:10.1093/gigascience/gix085)
Supplement: Response-to-Reviewer-Comments_Original-Submission.pdf [file gix085_Response-to-Reviewer-Comments_Original-Submission.pdf]

Author's Response To Reviewer Comments

Reviewer #1:

General Comments

The paper by Korlach et al describes the assembly of the zebrafinch and hummingbird genomes as well as an exhaustive analysis about the differences between a PacBio assembly and those of Sanger and Illumina. I never believed that short reads would produce reliable assemblies, and this paper shows in excruciating detail how many errors there are. At then end of this I want to declare RESEQUENCE and REANNOTATE all short-read genomes.

Response: Our sentiments are the same, and therefore the G10K and associated consortiums are just starting to do that (re-sequence many genomes with long reads) based in part on the results of this paper.

One of the advances in the study was the use of an assembler that produces a diploid assembly. Historically, computational biologists (or maybe computer scientists) have conveniently (for their purposes) posed the sequence assembly problem as the reconstruction of a haploid genome. Certainly, in the days of E. coli and S. cerevisiae this made sense, and also with the first eukaryotic genomes that were true-breeding laboratory strains. But vertebrates aren't haploid, and mashing a diploid genome with distinct haplotypes into a haploid genome is bound to cause problems. This paper is in a somewhat unique position to answer that question, but they don't.

Response: We thank the reviewer for this good point. In response to this concern, which the reviewer further highlighted below, we have now provided a Table 2 showing the types of errors in the prior data analyzed, what caused them, and what feature prevented them: long reads, SMRT enzyme read through of normally difficult-to-sequence regions, and/or haplotype phasing. We also further note that these three improvements are synergistic, meaning, for example, that reading through difficult regions helps improve longer reads, and both in turn allows for better assembly and phasing of haplotypes.

Specific Comments

1. Line 72 "an GC-rich"

Response: We have applied the correction to now read “a GC-rich”.

2. There are a lot of references to the PacBio genome being better than the Sanger and Illumina on a variety of metrics. It isn't clear to me how much of this is due to the diploid assembly and how much to the long reads. Is there some way of teasing these apart? I think so. They had a merged reference at one point. It would be interesting to see comparisons to that. I think people want to know how much of the improvement is expected to come from longer reads and how much will come from a diploid assembler. I understand that the two are somewhat linked, but some insight would be appreciated. To be clear on this point, do all the same analyses and add the PacBio haploid genome to the mix. Sorry, I know it's a big request.

Response: We apologize to the reviewer, in that we were not clear enough. We are able to answer these questions with existing data. The original FALCON assembly algorithm without the Unzip module is also diploid-aware, but only phases regions with highly divergent heterozygosity (Chin et al. (reference 18)). This resulted in unintentional indels and some structural errors in the merged regions, which we noted in our assemblies. We have included this information in Table 2, and cited Chin et al for the original findings.

For further details, the extended supplementary note of Chin et al. contains detailed figures of the differences between FALCON and FALCON-Unzip (Supplementary Figures 1, 10, 12, 13, 14 and 15 of Chin et al.) along with their corresponding text descriptions (pages 44-52). This also includes a discussion of how the different levels of heterozygosity affect the assembly string graphs and the contig layouts in FALCON and FALCON-Unzip (Supp. Fig. 10 and page 46). This complements the comparison of FALCON and FALCON-Unzip in the main Chin et al. paper (Table 1, Figure 1, text on first page right column bottom, second page bottom left column and top right column). Thus, the principal differences and examples of genome-wide comparisons between partially and mostly phased assemblies have already been published and are beyond the scope of this paper aimed to focus on the ramifications of higher assembly qualities for gene analyses.

However, following the reviewer's request we have added more detail in the Results section to indicate the general effects of Unzip module on the assemblies we generated. This includes the that FALCON-Unzip module yields higher contiguity for both haplotypes; in the case of the hummingbird, the contig N50 contig of the associated haplotype assembly changed from 40 kb to >1 Mb. We have also added a description of the ramifications of this for the gene examples studied in the paper, whereby smaller allelic structural variants that were not yet segregated by FALCON because their degree of divergence was below the threshold were successfully resolved by FALCON-Unzip.

3. How about a haplotype vs haplotype dot plot?

Response: We have included several examples of haplotype vs. haplotype dot plots (Fig. S10A, Fig. S14B, and also Fig. 6B in the form of an alignment view). We believe these examples, in conjunction with the overall assembly statistics, provide a good demonstration of the assembly output for given genes of interest. A genome-wide analysis of the degree and structure of heterozygosity over the entire genome is in our opinion beyond the scope of this paper, and is in fact targeted for a follow up study once these genomes have been scaffolded with optical mapping, Hi-C and other techniques, and the assemblies have been processed for a new reference genome release. This will then allow this type of analysis to be done on a chromosome-level scale.

4. Is it really necessary to dissect 4 genes? It seems too many or too few. What I'd rather see is 1 or 2 detailed dissections followed by a table showing the various kinds of problems and how often they occur (after having analyzed tens of genes).

Response: We think it is necessary to show the details for the 4 genes, as each has some unique

example feature that was corrected, that we have seen in dozens of genes. We think doing statistics on 10 random manually analyzed genes may still not be enough to do proper statistics, and doing many more genes would require an enormous amount of effort.

Reviewer #2:

General comments

This study is a nice illustration on how using longer reads can help disentangle difficult to sequence regions in eukaryote genomes, in particular two bird genomes. It also shows the power of software (in this case, FALCON and FALCON-Unzip) tailored towards assembling more than a single consensus haplotype. The authors describe in-depth comparisons between different assemblies of a selected set of challenging genes to successfully illustrate their main points. It is important to note that the lead author is Chief Scientific officer of the Pacific Biosciences company, but that the study appears to be a collaboration between the company and researchers from three academic groups.

Our main problem with this paper is the overconfidence that is placed in the PacBio data and the assemblies generated from it. Often, the paper reads as promotional material for the Pacific Biosciences technology. We would ask the authors to remain open for the fact that the PacBio based assemblies are not perfect and could contain errors, and reflect that in the text.

For example, the authors write several times about "sequence that was (not) supported by the PacBio data", e.g. four times on page 7 (lines 21-224 and 244), as well as on page 10 line 343. This gives the impression that the PacBio data is the gold standard, and contributes to the 'sales pitch' feeling we get when reading the manuscript.

Response: We are glad that the reviewer liked the study. We have revised the terminology so that it does not appear promotional, which was not our intention. We still used the terms Sanger-based, Illumina-based, and Pacbio-based assemblies, as opposed to medium-, short-, and long-read based assemblies, in part because there are more differences between these technologies and associated algorithms for assembly than read length. We now mention this in the main text. We also now further highlight that in addition to the indel errors already mentioned in the mostly unphased assembly, there could be possible errors in the Pacbio-based phased assemblies that we have not detected, because the other technologies may not have been able to sequence those regions for us to compare with. We also now replace the term “supported” with not present or present in the independent data. Finally, reviewer #1 asked us to put a table (Table 2) that shows what types of errors the PacBio-based assemblies prevented. In that table, we also include the indel and misplaced repeat sequence errors that occurs with the PacBio-based FALCON (i.e. mostly unphased and merged) assembly.

In several places, the assembly is validated using the long reads used to generate it, as well as PacBio-sequencing-based transcriptome evidence. This means these validations are done against PacBio data, and thus are only as useful and valid within that setting - one should not assume that the PacBio data is fully error free, even if so far no systematic biases have been detected with this data, nor that the assembly algorithm is flawless.

Response: We have added a statement that these validations are done against the PacBio data, even if no systematic error bias has been noted thus far in the data we used. We note that some of the validations were done with Illumina RNA-Seq data on the Pacbio assembly, and therefore are not subject to this concern.

The statement on page 13, line 458 "Thus far, we have not seen an error (i.e. difference) in the genes we examined in the PacBio-based long-read assembly relative to the other assemblies that was supported by single sequenced genomic DNA molecules, RNA-Seq and Iso-Seq mRNA molecules, or other independent evidence" at least includes non-PacBio evidence, such as comparing the genes in the focus set to genes from other species. But the lack of orthogonal data means there is no other evidence to more extensively validate the PacBio-bases assemblies. The authors could have compared to other independent evidence, such as finished BACs (zebra finch) or long-insert mate pair libraries, such as an Illumina 20 kbp library (Anna's hummingbird). Showing improvements using these data would strengthen the trust in the new assemblies considerably.

Response: We consider the RNA-Seq and Iso-Seq data orthogonal and therefore disagree that there is lack of orthogonal data. With regard to the reviewer's request for additional orthogonal data, several of the authors tested generating a single zebra finch BAC with the EGR1 gene many years ago using the Sanger method, but were unable to get through the GC-rich promoter region as described here. The 20 kb mate pair libraries are not orthogonal to the Illumina-based assembly as they were used to generate the Illumina-based assemblies, and are generated with short reads. However, following the reviewer's request, we have now included analyses of orthogonal single Sanger-based sequenced clones we previously published on the DUSP1 gene. Consistent with our other findings, the single clone Sanger-based sequences and assembly support the PacBio-based genome assembly, and not the Sanger-based genome assembly. We have added this result as a new supplementary Figure S11.

In general, the PacBio-based assembly are _improvements_ over the short-read and Sanger-based assemblies, but not necessarily always _corrections_ of them, and the manuscript should reflect that interpretation to a larger degree

Response: We believe that the above mentioned revisions now communicate this message, that the Pacbio-based assemblies are improvements, but still subject to possible errors.

The authors confuse sequence identity, which can be expressed as a percentage, with sequence homology, which is either the case or not. E.g. on page 7 line 236 "with high (93%) sequence homology"; 'homology' should be 'identity' here.

Response: We changed homology to identity, and have made the same changes in two other like it. We thank the reviewer for noting this.

In an attempt to verify some of the findings, it became clear that it would help to have a list genBank IDs for the relevant contigs, and a list of which regions on the PacBio contigs corresponds to the genes selected for detailed analysis. For example, we had to deduce that for T.

guttate, contig 32 is GenBank entry MUGN01001067, and, from Supplementary figure S8, that the region containing the UDSP1 gene was from bp 1761454 to 1769224 on the reverse complement, (which we had missed at first, leading us to take the region from the original commit, resulting in a blast match in a very different region). When we had the correct sequence, we could map it to the Zebra finch assembly in UCSC and confirm the relevant findings in figure 4.

Time constraints have prevented us from performing further validation experiments (assembolies, mapping of genomic DNA, RNAseq og CHIP-seq reads).

Response: We are glad that the reviewers were able to replicate our findings with their own analyses. Following the reviewer's request, we have noted the location of the contig label translation tables in NCBI in the paper. We also now include the two additional files that list all the contig IDs and their associated NCBI contig designations, including those mentioned in the paper, in the form of two supplementary tables for the zebra finch and hummingbird, respectively (Tables S1 & S2).

Minor comments

(line numbers used in the following are those added by the authors, not those from the editorial management system)

Abstract line 32, "However, often genes of interest are not completely or accurately assembled," is an overgeneralisation, please replace with "However, genes of interest are sometimes not completely or accurately assembled"

Response: We have made the change to "sometimes".

Table 1: Can the authors comment on the fact that the total sizes for the PacBio-based primary haplotype sequences are shorter than for the reference assemblies?

Response: The detailed analysis of this is currently being undertaken as part of the new zebra finch reference generation, incorporating the current reference, PacBio assembly, scaffolding techniques, RNA- and Iso-Seq data, and other datasets. While we cannot rule out that there are regions missing in the PacBio assembly, haploid reference genomes often contain unplaced contigs/scaffolds that in fact represent alternative haplotypes. In addition, in some cases the alternative haplotype is erroneously incorporated into the haploid reference genome representation, as illustrated for DUSP1 in the manuscript. Both of these effects tend to result in an overestimate of the haploid genome size in the Illumina-based short-read or Sanger-based intermediate-read reference genomes.

Table 1, I don't understand this: "The higher number of contigs in the secondary haplotype (5th column) are a result of the arbitrary assignment of shorter haplotypes to the haplotig category."

Response: We further explained this result in the revised manuscript, being that the longest contig is chosen for the primary haplotype, whether it is from the maternal or paternal

chromosome, since the later information is not known. This is also described in detail in Chin et al. (reference 18, Figure 1C), we have added the reference to help clarify this assembly algorithm principle.

page 2 line 58 'costly' in relation to the Sanger method is unnecessary, please remove

Response: We have removed the word ‘costly’, and replaced with ‘original’.

p.2 l.62 "With the advent of more cost-effective next generation sequencing technologies using short reads, 10-fold more genomes were sequenced," What is this '10-fold' based on?

Response: This refers to the G10K consortium paper by O’Brien et al 2015, we have added the reference and added ‘vertebrate’ to this statement.

p.2 l.66 It was a surprise to learn that the Avian Phylogenomics Consortium sequenced several reptiles, and it would help to refer to https://urldefense.proofpoint.com/v2/url?u=http-3A__science.sciencemag.org_content_346_6215_1254449&d=DwIGaQ&c=JeTkUgVztGMmhKYjxsy2rfoWYibK1YmxXez1G3oNStg&r=WGlElfIPa5SWQNtpBtGxpU2oIEw0BKiJJYXmODNuJNE&m=_1Tpbr3I6s-K2m5IWhd11iE8sg6GVQiEIDCwMGBNaEs&s=mpGPC4Ixqd92WQAzCoOpDfsxe6VNPUBWOCnXteBwqco&e= here.

Response: This paper has now been cited.

p.5 l. 155 "When we manually examined genes randomly" we suspect the authors mean "When we manually examined randomly chosen genes"

Response: This correction has been made.

p. 5 l 171 When mapping is done to the PacBio assembly, is it to the primary haplotigs, or to both primary and secondary haplotigs?

Response: The RNA-Seq and Chip-Seq mapping was done against the primary haplotigs, which we now state in the revised manuscript.

p. 6 lines 191-194 We don't follow the explanation for the "10% increase in unique mapped reads with a concomitant decrease in multiple mapped reads"

Response: To explain this difference between the RNA-Seq and Chip-Seq results, we revised the sentences to state: The RNA-Seq data was paired-end reads mapped to the genome, whereas the ChIP-Seq data was single-end reads; when just using the single-ends of the RNA-Seq data, the multiple-mapped increase to the Pacbio-based assembly was not detected (p=0.3, paired t-test, n=5), indicating that repetitive sequence may be in the paired end data that influences read mapping

p. 8 l. 290 and 292 mention R' and R", but the figure uses R1', R2', R1" and R2"

Response: We have clarified this in the text by adding the appropriate numbers to the R designations in the text.

p. 9 l. 299 Fig 10A —> Fig. S10A

Response: Change made.

p9. l. 334 "yet we find that the very large gene body of ~400 kb is incompletely assembled, including in vocal learning species " is FOXP2 _always_ incompletely assembled, in every species studied? If not, please rephrase ('often'?)

Response: It is incompletely assembled in all the species examined. So, we removed the statement "including in vocal learning species".

p 11. l. 407 "The two alleles were phased along the entire length of the gene." Could the full read alignment be shown in the supplementary to support this statement?

Response: Since this is implicit in the earlier statement of the resolution of SLIT1 into the two allelic contigs, we have added "and phased" to that earlier statement and removed the sentence. We could show the section of the entire gene region, but a dot plot would not show the heterozygous SNPs since the gene is so large, and an alignment file would also be very large not showing the few hetSNPs clearly. We feel that the combination of designating the two alleles with the two separate contigs, and explicitly showing the two haplotype sequences in Fig. 6B are adequate to make this point.

Methods: It would help the reader who may be interested in reusing some of your methods if complete command lines were made available, e.g. for the assembly, mapping, Augustus gene prediction etc.

Response: Since these are published software pipelines we feel that appropriate citations thereto are sufficient.

p. 14 l. 507 FALCON and FALCON-Unzip: It would be advantageous if there was a dedicated release of this software available on github (from
https://urldefense.proofpoint.com/v2/url?u=https-
3A__github.com_PacificBiosciences_FALCON_releases&d=DwIGaQ&c=JeTkUgVztGMmhK
Yjxsy2rfoWYibK1YmxXez1G3oNStg&r=WGlElfIPa5SWQNtpBtGxpU2oIEw0BKiJJYXmOD
NuJNE&m=_1Tpbr3I6s-
K2m5IWhd11iE8sg6GVQiEIDCwMGBNaEs&s=fg4j7QZnEWc8JVkG7VNi2_vPfpeUTC5Nar
xnZ8RQr_g&e=)

Response: FALCON-Unzip is available on github, as referenced in Chin et al. (reference 16 in the paper, https://github.com/PacificBiosciences/FALCON_unzip), cited in the appropriate location. We also now list in it in the current paper.

p. 14 l. 527 Why the was only the human CEGMA gene set used?

Response: The human gene set is the phylogenetically closest set, since all other CEGMA gene sets are from non-vertebrates (http://korflab.ucdavis.edu/datasets/cegma/). This is also been added to the text (in methods).

p. 14 l. 529 why was genblastA used and not the CEGMA software for the mapping?

Response: The CEGMA software is no longer actively supported and we have had good results using genblastA for mapping the genes onto the assemblies, and outputs allowing further processing (e.g. with exonerate).

p. 14 l. 530 please make these "custom shell scripts" available

Response: For genblastA, we used the command: genblast_v138_linux_x86_64 -p genblasta -t ${genome} -q ${CEGMA_genes} -c 0.3 -e 0.00001 -gff -pid -r 1, where ${genome} is the assembly and ${CEGMA_genes} is the CEGMA file; the output file contains the alignment percentage for each gene. For exonerate, we used the command: exonerate -m protein2genome --percent 30 --bestn 1 --showtargetgff --ryo ">%qi\n%tcs\n%m\n" -q CEGMA_prot.fa -t contig.fa, where CEGMA_prot.fa is a CEGMA peptide and contig.fa is the corresponding contig in the assembly. The vulgar output of this command (F designator) contains the number of frame shifts per gene. We have added this information to the text.

p. 14 l. 537/538 "We then ran a one-sided paired Wilcoxon signed-rank test of the two lengths for each protein. " How was this done? Please make the code to do this available if appropriate.

Response: It was performed in R, using the “wilcox.test” function with “paired = T.” The information has been added to the text.

p. 15 l. 560 "nine adult male zebra finches were isolated in soundproof chambers" I suspect that the authors meant "zebra finches were kept isolated in soundproof chambers"

Response: Correction made

p. 15 l 595/595 Which versions of Blasr and GMAP were used with that settings?

Response: We used Blasr version 3.1.0 and GMAP version 2016-08-16, respectively; this has been added to the text.

Figure legends:

Fig 1, p. 18 l. 642 Please explain why "We used a more stringent cut-off (> 95%) for completeness than usually done (> 90%)."

Response: We used the more stringent cut off because we felt 90% was too arbitrary and can allow entire missing exons and still call the gene complete. This is now mentioned in the figure

legend.

Fig 1, p. 18 l. 642 Gent —> Gene
Response: Correction made
