# Supplementary material for: De novo PacBio long-read and phased avian genome assemblies correct and add to reference genes generated with intermediate and short reads
Source: Gigascience. 2017 Aug 28;6(10):1–16. doi: 10.1093/gigascience/gix085 (PMC5632298; doi:10.1093/gigascience/gix085)
Supplement: Reviewer-1-Report-(Original-Submission).pdf [file gix085_Reviewer-1-Report-(Original-Submission).pdf]

Reviewer Report

Title: De Novo PacBio long-read and phased avian genome assemblies correct and add to reference genes generated with intermediate and short reads

Version: Original Submission **Date:** 4/3/2017

Reviewer name: Ian Korf

Reviewer Comments to Author:

General Comments

The paper by Korlach et al describes the assembly of the zebrafinch and hummingbird genomes as well as an _exhaustive_ analysis about the differences between a PacBio assembly and those of Sanger and Illumina. I never believed that short reads would produce reliable assemblies, and this paper shows in _excruciating_ detail how many errors there are. At then end of this I want to declare RESEQUENCE and REANNOTATE all short-read genomes.

One of the advances in the study was the use of an assembler that produces a diploid assembly. Historically, computational biologists (or maybe computer scientists) have conveniently (for their purposes) posed the sequence assembly problem as the reconstruction of a haploid genome. Certainly, in the days of E. coli and S. cereviseae this made sense, and also with the first eukaryotic genomes that were true-breeding laboratory strains. But vertebrates aren't haploid, and mashing a diploid genome with distinct haplotypes into a haploid genome is bound to cause problems. This paper is in a somewhat unique position to answer that question, but they don't.

Specific Comments

1. Line 72 "an GC-rich"

2. There are a lot of references to the PacBio genome being better than the Sanger and Illumina on a variety of metrics. It isn't clear to me how much of this is due to the diploid assembly and how much to the long reads. Is there some way of teasing these apart? I think so. They had a merged reference at one point. It would be interesting to see comparisons to that. I think people want to know how much of the improvement is expected to come from longer reads and how much will come from a diploid assembler. I understand that the two are somewhat linked, but some insight would be appreciated. To be clear on this point, do all the same analyses and add the PacBio haploid genome to the mix. Sorry, I know it's a big request.

3. How about a haplotype vs haplotype dot plot?

4. Is it really necessary to dissect 4 genes? It seems too many or too few. What I'd rather see is 1 or 2

detailed dissections followed by a table showing the various kinds of problems and how often they occur (after having analyzed tens of genes).

Methods

Are the methods appropriate to the aims of the study, are they well described, and are necessary controls included? Yes

Conclusions

Are the conclusions adequately supported by the data shown? Yes

Reporting Standards

Does the manuscript adhere to the journal's guidelines on minimum standards of reporting? YesChoose an item.

Statistics

Are you able to assess all statistics in the manuscript, including the appropriateness of statistical tests used? Yes, and I have assessed the statistics in my report.

Quality of Written English

Please indicate the quality of language in the manuscript: Acceptable

Declaration of Competing Interests

Please complete a declaration of competing interests, considering the following questions:

- Have you in the past five years received reimbursements, fees, funding, or salary from an organisation that may in any way gain or lose financially from the publication of this manuscript, either now or in the future?
- Do you hold any stocks or shares in an organisation that may in any way gain or lose financially from the publication of this manuscript, either now or in the future?
- Do you hold or are you currently applying for any patents relating to the content of the manuscript?
- Have you received reimbursements, fees, funding, or salary from an organization that holds or has applied for patents relating to the content of the manuscript?
- Do you have any other financial competing interests?
- Do you have any non-financial competing interests in relation to this paper?

If you can answer no to all of the above, write 'I declare that I have no competing interests' below. If your reply is yes to any, please give details below.

I declare that I have no competing interests

I agree to the open peer review policy of the journal. I understand that my name will be included on my report to the authors and, if the manuscript is accepted for publication, my named report including any attachments I upload will be posted on the website along with the authors' responses. I agree for my report to be made available under an Open Access Creative Commons CC-BY license (http://creativecommons.org/licenses/by/4.0/). I understand that any comments which I do not wish to be included in my named report can be included as confidential comments to the editors, which will not be published.

I agree to the open peer review policy of the journal

To further support our reviewers, we have joined with Publons, where you can gain additional credit to further highlight your hard work (see: https://publons.com/journal/530/gigascience). On publication of this paper, your review will be automatically added to Publons, you can then choose whether or not to claim your Publons credit. I understand this statement.

Yes
