# Supplementary material for: De novo PacBio long-read and phased avian genome assemblies correct and add to reference genes generated with intermediate and short reads
Source: Gigascience. 2017 Aug 28;6(10):1–16. doi: 10.1093/gigascience/gix085 (PMC5632298; doi:10.1093/gigascience/gix085)
Supplement: Reviewer-2-Report-(Original-Submission).pdf [file gix085_Reviewer-2-Report-(Original-Submission).pdf]

**Reviewer Report**

**Title:** De Novo PacBio long-read and phased avian genome assemblies correct and add to reference genes generated with intermediate and short reads

**Version:** Original Submission **Date:** 4/6/2017

**Reviewer name:** Lex Nederbragt

**Reviewer Comments to Author:**

This study is a nice illustration on how using longer reads can help disentangle difficult to sequence regions in eukaryote genomes, in particular two bird genomes. It also shows the power of software (in this case, FALCON and FALCON-Unzip) tailored towards assembling more than a single consensus haplotype. The authors describe in-depth comparisons between different assemblies of a selected set of challenging genes to successfully illustrate their main points. It is important to note that the lead author is Chief Scientific officer of the Pacific Biosciences company, but that the study appears to be a collaboration between the company and researchers from three academic groups.

Our main problem with this paper is the overconfidence that is placed in the PacBio data and the assemblies generated from it. Often, the paper reads as promotional material for the Pacific Biosciences technology. We would ask the authors to remain open for the fact that the PacBio based assemblies are not perfect and could contain errors, and reflect that in the text.

For example, the authors write several times about "sequence that was (not) supported by the PacBio data", e.g. four times on page 7 (lines 21-224 and 244), as well as on page 10 line 343. This gives the impression that the PacBio data is the gold standard, and contributes to the 'sales pitch' feeling we get when reading the manuscript.

In several places, the assembly is validated using the long reads used to generate it, as well as PacBio-sequencing-based transcriptome evidence. This means these validations are done against PacBio data, and thus are only as useful and valid within that setting - one should not assume that the PacBio data is fully error free, even if so far no systematic biases have been detected with this data, nor that the assembly algorithm is flawless.

The statement on page 13, line 458 "Thus far, we have not seen an error (i.e. difference) in the genes we examined in the PacBio-based long-read assembly relative to the other assemblies that was supported by single sequenced genomic DNA molecules, RNA-Seq and Iso-Seq mRNA molecules, or other independent evidence" at least includes non-PacBio evidence, such as comparing the genes in the focus set to genes from other species. But the lack of orthogonal data means there is no other evidence to more extensively validate the PacBio-bases assemblies. The authors could have compared to other independent evidence, such as finished BACs (zebra finch) or long-insert mate pair libraries, such as an Illumina 20 kbp library (Anna's hummingbird). Showing improvements using these data would

strengthen the trust in the new assemblies considerably.

In general, the PacBio-based assembly are _improvements_ over the short-read and Sanger-based assemblies, but not necessarily always _corrections_ of them, and the manuscript should reflect that interpretation to a larger degree

The authors confuse sequence identity, which can be expressed as a percentage, with sequence homology, which is either the case or not. E.g. on page 7 line 236 "with high (93%) sequence homology"; 'homology' should be 'identity' here.

In an attempt to verify some of the findings, it became clear that it would help to have a list genBank IDs for the relevant contigs, and a list of which regions on the PacBio contigs corresponds to the genes selected for detailed analysis. For example, we had to deduce that for T. guttate, contig 32 is GenBank entry MUGN01001067, and, from Supplementary figure S8, that the region containing the UDSP1 gene was from bp 1761454 to 1769224 on the reverse complement, (which we had missed at first, leading us to take the region from the original commit, resulting in a blast match in a very different region). When we had the correct sequence, we could map it to the Zebra finch assembly in UCSC and confirm the relevant findings in figure 4.

Time constraints have prevented us from performing further validation experiments (assembolies, mapping of genomic DNA, RNAseq og CHIP-seq reads).

Minor comments

(line numbers used in the following are those added by the authors, not those from the editorial management system)

Abstract line 32, "However, often genes of interest are not completely or accurately assembled," is an overgeneralisation, please replace with "However, genes of interest are sometimes not completely or accurately assembled"

Table 1: Can the authors comment on the fact that the total sizes for the PacBio-based primary haplotype sequences are shorter than for the reference assemblies?

Table 1, I don't understand this: "The higher number of contigs in the secondary haplotype (5th column) are a result of the arbitrary assignment of shorter haplotypes to the haplotig category."

page 2 line 58 'costly' in relation to the Sanger method is unnecessary, please remove

p.2 l.62 "With the advent of more cost-effective next generation sequencing technologies using short

reads, 10-fold more genomes were sequenced," What is this '10-fold' based on?

p.2 l.66 It was a surprise to learn that the Avian Phylogenomics Consortium sequenced several reptiles, and it would help to refer to http://science.sciencemag.org/content/346/6215/1254449 here.

p.5 l. 155 "When we manually examined genes randomly" we suspect the authors mean "When we manually examined randomly chosen genes"

p. 5 l 171 When mapping is done to the PacBio assembly, is it to the primary haplotigs, or to both primary and secondary haplotigs?

p. 6 lines 191-194 We don't follow the explanation for the "10% increase in unique mapped reads with a concomitant decrease in multiple mapped reads"

p. 8 l. 290 and 292 mention R' and R'', but the figure uses R1', R2', R1'' and R2''

p. 9 l. 299 Fig 10A —> Fig. S10A

p9. l. 334 "yet we find that the very large gene body of ~400 kb is incompletely assembled, including in vocal learning species " is FOXP2 _always_ incompletely assembled, in every species studied? If not, please rephrase ('often'?)

p 11. l. 407 "The two alleles were phased along the entire length of the gene." Could the full read alignment be shown in the supplementary to support this statement?

Methods: It would help the reader who may be interested in reusing some of your methods if complete command lines were made available, e.g. for the assembly, mapping, Augustus gene prediction etc.

p. 14 l. 507 FALCON and FALCON-Unzip: It would be advantageous if there was a dedicated release of this software available on github (from https://github.com/PacificBiosciences/FALCON/releases)

p. 14 l. 527 Why the was only the human CEGMA gene set used?

p. 14 l. 529 why was genblastA used and not the CEGMA software for the mapping?

p. 14 l. 530 please make these "custom shell scripts" available

p. 14 l. 537/538 "We then ran a one-sided paired Wilcoxon signed-rank test of the two lengths for each protein. " How was this done? Please make the code to do this available if appropriate.

p. 15 l. 560 "nine adult male zebra finches were isolated in soundproof chambers" I suspect that the authors meant "zebra finches were kept isolated in soundproof chambers"

p. 15 l 595/595 Which versions of Blasr and GMAP were used with that settings?

Figure legends:

Fig 1, p. 18 l. 642 Please explain why "We used a more stringent cut-off (> 95%) for completeness than usually done (> 90%)."

Fig 1, p. 18 l. 642 Gent —> Gene

------

Reviewed by Lex Nederbragt and Ole Kristian Tørresen, Dept. of Biosciences, University of Oslo

**Methods**

Are the methods appropriate to the aims of the study, are they well described, and are necessary controls included? Yes

**Conclusions**

Are the conclusions adequately supported by the data shown? Yes

**Reporting Standards**

Does the manuscript adhere to the journal's guidelines on minimum standards of reporting? YesChoose an item.

**Statistics**

Are you able to assess all statistics in the manuscript, including the appropriateness of statistical tests used? No, and I do not feel adequately qualified to assess the statistics.

**Quality of Written English**

Please indicate the quality of language in the manuscript: Acceptable

**Declaration of Competing Interests**

Please complete a declaration of competing interests, considering the following questions:

- Have you in the past five years received reimbursements, fees, funding, or salary from an organisation that may in any way gain or lose financially from the publication of this manuscript, either now or in the future?

- Do you hold any stocks or shares in an organisation that may in any way gain or lose financially from the publication of this manuscript, either now or in the future?
- Do you hold or are you currently applying for any patents relating to the content of the manuscript?
- Have you received reimbursements, fees, funding, or salary from an organization that holds or has applied for patents relating to the content of the manuscript?
- Do you have any other financial competing interests?
- Do you have any non-financial competing interests in relation to this paper?

If you can answer no to all of the above, write 'I declare that I have no competing interests' below. If your reply is yes to any, please give details below.

1. Pacific Biosciences, represented with two authors on this manuscript, have contributed with data generated for free by the company to past research that we were involved with.

I agree to the open peer review policy of the journal. I understand that my name will be included on my report to the authors and, if the manuscript is accepted for publication, my named report including any attachments I upload will be posted on the website along with the authors' responses. I agree for my report to be made available under an Open Access Creative Commons CC-BY license (http://creativecommons.org/licenses/by/4.0/). I understand that any comments which I do not wish to be included in my named report can be included as confidential comments to the editors, which will not be published.

I agree to the open peer review policy of the journal

To further support our reviewers, we have joined with Publons, where you can gain additional credit to further highlight your hard work (see: https://publons.com/journal/530/gigascience). On publication of this paper, your review will be automatically added to Publons, you can then choose whether or not to claim your Publons credit. I understand this statement.

Yes
