# Supplementary material for: De novo PacBio long-read and phased avian genome assemblies correct and add to reference genes generated with intermediate and short reads
Source: Gigascience. 2017 Aug 28;6(10):1–16. doi: 10.1093/gigascience/gix085 (PMC5632298; doi:10.1093/gigascience/gix085)
Supplement: Additional Files [file gix085_Supp.zip › Supplementary Figures Legend.doc]

**Supplementary Figure S1.** DNA isolation, library construction, and size selection. *(A)* Pulsed-field gel showing original size of starting genomic DNA (lane 3), the sheared DNA (1), and the size selected library (2). *(B)* Bioanalyzer trace before (blue) and after (red) library size selection for fragments > 17 kb.

**Supplementary Figure S2.** Read and insert length distributions. *(A, B)* Sequence read length distributions from SMRT cell sequencing for both species. *(C, D)* Sequenced DNA insert length distributions from SMRT cell sequencing for both species.

**Supplementary Figure S3.** Box plots comparing protein coding sequence lengths of orthologous proteins between the CEGMA and BUSCO eukaryotic and avian datasets. ** p < 0.001; *** p < 0.0001, one-sided paired Wilcoxon signed-rank test, prediction of the proteins being longer in CEGMA datasets.

**Supplementary Figure S4.** Vocal learning and adjacent brain regions in songbirds used for RNA-Seq and ChIP-Seq analyses, and comparison with humans. *(A)* Drawing of a zebra finch male brain section showing specialized vocal learning pathway and associated profiled song nuclei RA, HVC, LMAN, and Area X. *(B)* Drawing of a human brain section showing spoken-language pathway and analogous brain regions. Black arrows, posterior vocal motor pathway; White arrows, anterior vocal learning pathway; Dashed arrows, connections between the two pathways; Red arrow, specialized direct projection from forebrain to brainstem vocal motor neurons in vocal learners. Italicized letters adjacent to the song and speech regions indicates regions (in songbirds) that show mainly show motor *(m)*, auditory *(a)*, equally both motor and auditory *(m/a)* neural activity or activity-dependent gene expression. Figure from [59] and [4].

*Abbreviations*: A1-L4, primary auditory cortex – layer 4; Am, nucleus ambiguous; Area X, a vocal nucleus in the striatum; aSt, anterior striatum vocal region; aT, anterior thalamus speech area; Av, avalanche; aDLM, anterior dorsolateral nucleus of the thalamus; DM, dorsal medial nucleus of the midbrain; HVC, a vocal nucleus (no abbreviation); L2, auditory area similar to human cortex layer 4; LSC, laryngeal somatosensory cortex; LMC, laryngeal motor cortex; MAN, magnocellular nucleus of the anterior nidopallium; MO, oval nucleus of the anterior mesopallium; NIf, interfacial nucleus of the nidopallium; PAG, peri-aqueductal gray; RA, robust nucleus of the arcopallium; v, ventricle space

**Supplementary Figure S5.** Dot plot of sequence comparisons for genome assemblies of the *EGR1* region. *(A)* Comparison of zebra finch PacBio-based versus Sanger-based assemblies for the region containing *EGR1*, showing the GC-rich promoter region and closing and corrections of gaps for the PacBio-based assembly. *(B)* Comparison of hummingbird Illumina-based versus PacBio-based assemblies for the region containing *EGR1*, showing an erroneous tandem duplication in the Ilumina-based assembly and closing of gaps for the PacBio-based assembly.

**Supplementary Figure S6.** Single SMRT genomic reads and Iso-Seq mRNA reads supporting Pacbio *EGR1* assembly. *(A)* Zebra finch PacBio SMRT reads (rows) mapped against the zebra finch PacBio assembly (contig 405, entire *EGR1* region, same as Fig. 3A). Reads are shaded by length (>10 kb reads = black). *(B)* Example of a single Ruby-throated hummingbird Iso-Seq read mapped against Illumina-based (top) and PacBio-based (bottom) Anna’s hummingbird genome assemblies using GMAP. Note the first exon (blue) which is present in the Iso-Seq read is missing in the Illumina-based assembly, but present in the PacBio-based assembly.

**Supplementary Figure S7.** Dot plot of sequence comparison for the PacBio-based hummingbird and zebra finch *EGR1* region assemblies. Note regions of high species conservation and divergence surrounding *EGR1*. Blue box, location of the *EGR1* exons and intron.

**Supplementary Figure S8.** Dot plot comparisons for *DUSP1* region assemblies. *(A)* Comparison of the Sanger-based and PacBio-based zebra finch *DUSP1* region assemblies, showing problems in the Sanger-based assembly with microsatellite repeats. *(B)* Comparison of the Illumina-based and PacBio-based hummingbird *DUSP1* region assemblies, showing a large gap including the microsatellite region and the beginning of the gene, and an erroneous tandem duplication in the Illumina-based assembly.

**Supplementary Figure S9.** Pacbio correction of base call errors found in Sanger reference *(A)* Confirmation of the PacBio sequence in the three locations different from the zebra finch Sanger reference by alignments to DUSP1 sequences of other songbirds. *(B)* PacBio reads (rows) corresponding to the genomic region in DUSP1 that differs in the three locations from the zebra finch Sanger reference, resulting in a.a. changes. The codons in question are highlighted.

**Supplementary Figure S10.** Dot plot comparison of assemblies for the *DUSP1* microsatellite region. *(A)* Differences in the microsatellite region upstream of the *DUSP1* protein coding sequence between the primary and the secondary haplotypes in the fully assembled zebra finch PacBio-based assembly. *(B)* Differences in microsatellites region upstream of *DUSP1* between the zebra finch and hummingbird in the fully assembled PacBio-based assemblies.

**Supplementary Figure S11.** Dot plot comparisons for PacBio-based *DUSP1* region assemblies with orthogonal validation. Comparison of the PacBio-based genome assembly and Sanger-based single clone of the *(A)* zebra finch and *(B)* hummingbird *DUSP1* upstream region assemblies showing more consistency between the two (than in **Fig S8A**). Not visible in this high-level alignment view is an 11-bp deletion and several SNPs in this allele of the PacBio contig relative to the other allele; the single clone of the individual is more consistent with the alternate allele without the 11-bp deletion.

**Supplementary Figure S12.** Single Iso-Seq mRNA reads supporting Pacbio assemblies. *(A)* Full-length PacBio mRNA sequence Iso-Seq ruby throated hummingbird reads for DUSP1 aligned against the exons of the corresponding primary contigs from Anna’s hummingbird Illumina (top panel) and PacBio (bottom panel) assemblies. *(B)* Similar alignments for FOXP2 IsoSeq reads.

**Supplementary Figure S13.** Dot plot comparison of assemblies for the *FOXP2* region. *(A)* zebra finch, *(B)* hummingbird.

**Supplementary Figure S14.** *(A)* Multiple sequence alignment of the FOXP2 protein for the four assemblies (two zebra finch and two hummingbird) compared in this study, showing correction of a nucleotide error in the Sanger-based zebra finch assembly, and correction of an erroneous stop codon (x) in the Illumina-based hummingbird assembly. Note an extra 18 a.a. stretch in the hummingbird sequence validated by gene prediction of both assemblies, that was not present in the zebra finch. *(B)* Missing 88bp of sequence in exon 6 of Illumina-based assembly. *(C)* Resolution of exon 6 in Pacbio-based assembly, also revealing a SNP.

**Supplementary Figure S15.** Large regional correction made by the PacBio diploid assembly. *(A)* Correction of an erroneous stretch of 462 bp in the first intron of *FOXP2* in the hummingbird Illumina assembly by the PacBio assembly. *(B)* Dot plot of haplotype variation in the *FOXP2* gene revealed by the PacBio diploid assembly: a 708 bp deletion in the secondary haplotype contig relative to the primary contig.

**Supplementary Figure S16.** Dot plot comparison of assemblies for the *SLIT1* region. *(A)* zebra finch, *(B)* hummingbird.
